# An Overview of Anticoagulant Drugs Pharmacology, Therapeutic Approaches, Limitations and Perspectives

**DOI:** 10.3390/pharmaceutics18020163

**Published:** 2026-01-26

**Authors:** Claudiu Morgovan, Adina Frum, Laurentiu Stoicescu, Anca Butuca, Carmen Maximiliana Dobrea, Anca Maria Arseniu, Adriana Aurelia Chis, Maria Lucia Muresan, Felicia Gabriela Gligor, Ioana Rada Popa Ilie, Steliana Ghibu

**Affiliations:** 1 Preclinical Department, Faculty of Medicine, “Lucian Blaga” University of Sibiu, 550169 Sibiu, Romania; claudiu.morgovan@ulbsibiu.ro (C.M.); adina.frum@ulbsibiu.ro (A.F.); carmen.dobrea@ulbsibiu.ro (C.M.D.); anca.arseniu@ulbsibiu.ro (A.M.A.); maria.muresan@ulbsibiu.ro (M.L.M.); felicia.gligor@ulbsibiu.ro (F.G.G.); 2 Department of Cardiology, Vth Medical Clinic, Faculty of Medicine, “Iuliu Haţieganu” University of Medicine and Pharmacy, 400139 Cluj-Napoca, Romania; 3 Association for Excellence in Pharmaceutical Education and Research, 550169 Sibiu, Romania; a.adriana.chis@gmail.com; 4 Department of Endocrinology, Faculty of Medicine, “Iuliu Haţieganu” University of Medicine and Pharmacy, 3-5 Louis Pasteur Street, 400349 Cluj-Napoca, Romania; ioana.ilie@umfcluj.ro; 5 Department of Pharmacology, Physiology and Pathophysiology, Faculty of Pharmacy, “Iuliu Haţieganu” University of Medicine and Pharmacy, 6A Louis Pasteur Street, 400349 Cluj-Napoca, Romania; stelianaghibu@yahoo.com

**Keywords:** coagulation factors, venous thromboembolism, anticoagulant drugs, unfractionated heparin, low molecular weight heparins, vitamin K antagonists, direct oral anticoagulants, medication errors, reversal anticoagulant agents

## Abstract

Coagulation is a physiological process necessary to achieve homeostasis. Many pathologies lead to spontaneous activation of the coagulation pathways and increase the risk of venous thrombosis (e.g., atrial fibrillation, orthopaedic surgery, cancer). Therefore, a lot of patients need anticoagulant drugs as preventive or curative treatment. In general, older molecules (unfractionated heparin, low-molecular-weight heparins, vitamin K antagonists) have good efficacy. Still, their adverse reactions, increased risk of bleeding, or difficult administration led to low adherence to treatment and had even limited their use. Recently, new molecules were authorised to improve patient adherence to treatment, mainly formulated for oral administration (e.g., dabigatran, rivaroxaban, apixaban, etc.). This therapeutic approach has a low risk of bleeding and does not require special monitoring by laboratory tests. Also, new anticoagulants for patients with heparin-induced thrombocytopenia (e.g., argatroban, lepirudin, bivalirudin, etc.) were obtained. Moreover, reversal agents for the new anticoagulant molecules used in overdoses or in situations where immediate cessation of the anticoagulant effect is required (e.g., emergency surgery) were studied, some of them being authorised on the pharmaceutical market. This narrative review aims to provide a pharmacological and therapeutic overview of anticoagulant drugs, underlining their implementation and limitations.

## 1. Introduction

Isolated for the first time from the liver of an experimental animal injected with peptone, the heparphosphatide was discovered in 1916 by Jay McLean. Further, the substance was named heparin (1918), and after purification, it was structurally confirmed to be a sulfated glycosaminoglycan (1935). Its presence was detected in the lungs. In 1935, heparin was injected for the first time in human volunteers, and in the 1940s, it was used as antithrombotic therapy [1].

At the end of the 19th century, farmers in the United States of America were confronted with haemorrhage in animals fed with sweet clover, due to the presence of the active principle dicumarol (3,3′-methylene-bis-(4-hydroxycoumarin). Dicumarol was identified in 1940 and used for the first time as an antithrombotic in 1941. Since 1944, it has been used as an antithrombotic in patients with myocardial infarction [1,2]. Another important discovery in the field of anticoagulant drugs (ACs) was made in 1956, when hirudin was isolated in pure crystalline form from the salivary glands of the leech (*Hirudo medicinalis*). Ancient Egyptian doctors used leeching to treat fever. Basically, the properties of the leech’s saliva were known in Egyptian culture from ancient times. Many years later, the Romans used leeches to purify their bodies of toxic substances. Furthermore, their anticoagulant properties have been exploited since the 18th century. Nowadays, genetic engineering is used to produce biosynthetic hirudin and hirudin analogues [3,4].

**Figure 1:**
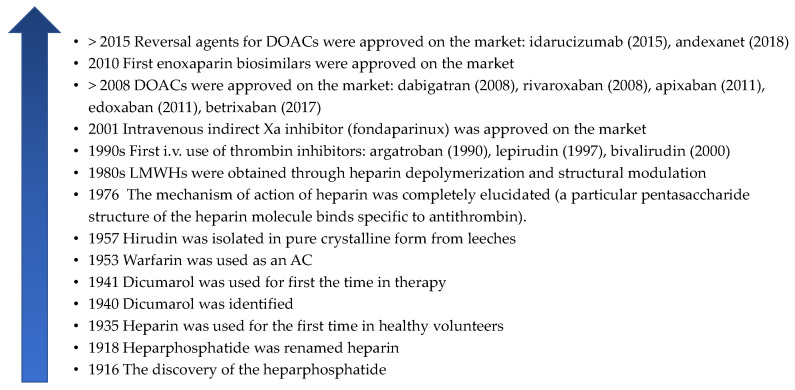
A brief history of anticoagulant drugs [1,2,3,4,6,8,11,12,13,14,15,17,18]. AC—anticoagulant drug, DOAC—direct oral anticoagulant drug.

Heparin’s mechanism of action was fully elucidated in 1976. Research has shown that a specific pentasaccharide unit in the structure of unfractionated heparin (UFH) specifically binds to an endogenous plasma glycoprotein, antithrombin III (ATIII), leading to the blocking of some of the coagulation factors (IIa, Xa, IXa, etc.) [5,6]. Subsequently, many low-molecular-weight heparins (LMWHs) obtained by depolymerisation of UFH were approved on the market: enoxaparin (1993), dalteparin sodium (1994), nadroparin calcium (1998), tinzaparin (1997), etc. Later, bemiparin sodium was the first ultra-low-molecular-weight heparin (ULMWH) approved, in 2006. Due to the positive evolution on the market, the competition has intensified, and since 2010, enoxaparin generics (biosimilars) have been used [7,8,9,10]. At that moment, a new era of anticoagulant drugs began, and other important discoveries were made. First, direct thrombin inhibitors with intravenous (i.v.) administration (argatroban, lepirudin, bivalirudin) were authorised. Then, fondaparinux, a synthetic small molecule that binds ATIII, was approved for the market in 2001. Since 2008, a new class of ACs has been launched, represented by direct oral anticoagulant drugs (DOACs), non-vitamin K antagonist oral anticoagulants (NOACs), or target-specific oral anticoagulants (TSOACs): dabigatran, rivaroxaban, apixaban, edoxaban, and betrixaban. The safety of DOACs was a primary objective for manufacturers. So, after only a few years, the first reversal agents for DOACs were synthesised (idarucizumab and andexanet) [6,11,12,13,14,15,16] (Figure 1).

Clinical practice directly drives market activity, as anticoagulant medications are dispensed solely by prescription. In 2017, ACs’ market value was 14% higher than in 2016. The growth of the ACs market value is justified, on the one hand, by the increased number of DOAC prescriptions and, on the other, by the difference between the average cost of treatment with warfarin (United States dollar—USD 20) and DOACs (USD 350) [19]. The total value of the DOACs market increased approximately four times, from USD 4.76 billion in 2013 to USD 17.07 billion in 2017 [19,20]. In 2021, the total value of the global ACs market was estimated at USD 28.6 billion [21]. The global market for ACs has an annual growth rate of 7.9%, and in 2027, the total value is estimated to become USD 41.87 billion [21]. Simultaneously with the growth of the oral ACs market, the ACs reversal agents’ market is also developing (USD 0.68 billion in 2019). Until 2027, the compound annual growth rate of these drugs is expected to be 14.3% [22].

Given this substantial increase in the number of ACs, the present study aimed to highlight the main pharmacological and therapeutic aspects of AC drugs. This manuscript provides an integrative up-to-date overview of traditional and newest generation anticoagulants. Pathophysiologic mechanisms, pharmacotherapeutic approaches, and clinical implications were analysed. Their integrative clinical–pharmacological perspective was highlighted, with particular focus on (i) anticoagulant management in special clinical conditions; (ii) clinical relevance of reversal agents; and (iii) real-world challenges associated with anticoagulant therapy, such as bleeding risk, administration difficulties, drug–drug interactions, medication errors, and treatment adherence. The review concludes by exploring future perspectives on anticoagulant development to improve the safety and efficacy of treatments.

## 2. The Coagulation Process and the Primary Targets of Anticoagulant Drugs

**Figure 2:**
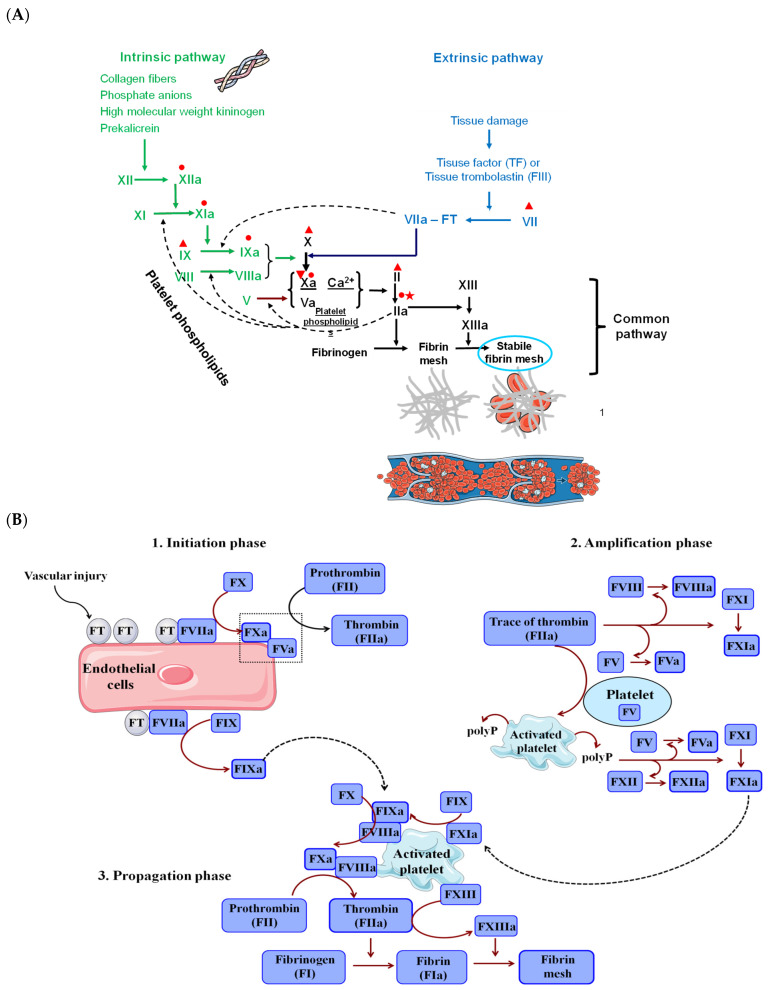
(**A**) Coagulation pathways and the anticoagulants’ primary targets. 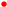
—UFH; 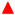
—vitamin K antagonists (VKAs); 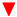
—LMWHs, ULMWHs, fondaparinux, DOACs; heparinoids (danaparoid); 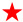
—LMWHs, ULMWHs, DOACs, hirudin, parenteral direct inhibitors of clotting factor IIa (ar-gatroban, desirudin, lepirudin, bivalirudin), heparinoids (danaparoid); (**B**) the three phases of the coagulation process [26,27,28,29,30].

Coagulation is a complex process that is part of physiological haemostasis (the plasma phase or the third phase of haemostasis), but is spontaneously activated under specific conditions, contributing to the development of venous thrombosis (VT) and, implicitly, to the complications arising from VT [23,24]. Thrombosis generally refers to the formation of blood clots within the veins or arteries, which partially or completely obstruct normal blood flow and are accompanied by clinical sequelae. Both venous and arterial thromboses have similar risk factors, such as age, obesity, smoking, chronic inflammation, metabolic syndrome, etc., and they represent the most frequent causes of mortality in developed countries. However, the pathophysiology, therapeutic approaches, and complications of the two types of thromboses differ [25]. Thus, VT typically begins with tissue damage and/or blood stasis. In contrast, arterial thrombosis originates from intimal surface irregularities (e.g., atherosclerosis or stent implantation) that facilitate platelet adhesion and aggregation. Therefore, the activation of coagulation factors and the formation of the fibrin mesh play a significant role in the development of venous thrombi, thereby explaining the use of anticoagulant agents in the prevention and treatment of this type of thrombosis [25]. To better understand the pathological implications of coagulation, especially the efficacy and differences among classes of anticoagulants, a brief review of the coagulation process is essential [23]. Thus, in the classical model, the key players of coagulation (coagulation cascade) are a series of plasma proteins (proenzymes) that are converted to active forms (active enzymes) by the upstream activated clotting factor and then ultimately lead to the transformation of soluble fibrinogen into the insoluble fibrin mesh, under the action of thrombin. In addition to plasma coagulation factors (clotting factors), plasma calcium, tissue thromboplastin (tissue factor, TF), and platelet phospholipids or the surface of activated platelets on which the coagulation factors need to be concentrated, also participate in this complex process [24]. To get to thrombin or the activated factor II (FIIa), there are two different pathways: (1) the intrinsic pathway, in which only plasmatic components participate, and (2) the extrinsic pathway that requires an external factor or the tissue factor, which is provided by some tissues and the vascular wall. These two pathways converge at the activation of factor X (FXa), and from this point forward, there is the common pathway of the coagulation cascade [24]. The intrinsic pathway is faster to activate in vitro when blood encounters hydrophilic or negatively charged surfaces and is more relevant for in vitro coagulation. This is triggered by autoactivated FXII (FXIIa), which cleaves prekallikrein into kallikrein and subsequently activates the factors FXI, FIX, FX, and prothrombin (Figure 2A). Subsequently, the extrinsic pathway is triggered by vascular or extravascular tissue injuries and is more important for in vivo coagulation. Thus, it starts with the tissue release of thromboplastin or TF, which binds to factor VII and activates it; once activated, factor VII rapidly induces the sequential activation of FX and ultimately the conversion of prothrombin (Figure 2A) [24].

According to a more recent approach or concept, the coagulation process is divided into three separated phases: (1) an initiation phase in which a low amount of coagulation factors is activated, (2) an amplification phase in which via some amplification loops in coagulation cascade, large amounts of fibrin are formed, and (3) a propagation phase in which the coagulation factors are concentrated on the phospholipids exposed in the platelet surface and stable fibrin meshes are formed (Figure 2B) [24]. An injury of the vessel walls classically induces the initiation phase, followed immediately by the exposure to the blood flow to thromboplastin or TF physiologically expressed by the fibroblasts or vascular smooth muscle fibres, and by the activation of factor VII (FVIIa) [23]. The factor VII activity is entirely dependent on the presence of the TF; TF binds to FVII, and thus the factor VII is activated [24]. Subsequently, this TF-VIIa complex initiates the coagulation cascade by activating both factor X and factor IX, mainly on the surface of the TF-expressing cells [24]. Once factor X is activated, together with its cofactor, activated factor V (FVa), they form a prothrombinase complex that cleaves prothrombin (FII) to its active form, thrombin (FIIa), thereby initiating the phase of coagulation [23]. FXa may dissociate from the TF-expressing cells to form prothrombinase complexes on cell membranes at other sites. However, the presence of protease inhibitors in plasma, such as serine protease inhibitor antithrombin (AT), can limit such diffusion. Nevertheless, FIXa is less susceptible to plasma proteases and can therefore diffuse more easily to other cell surfaces and participate in the propagation phase of coagulation [24]. In the amplification phase, the trace amount of thrombin (FIIa) slowly accumulates during the initiation phase. It activates factor XI, factor VIII (a cofactor for factor IXa), and factor V (a cofactor for factor Xa) in their active forms [24], thus significantly increasing the catalytic activity of FIX and FX [24]. In addition, thrombin can activate the platelets and stimulate the release of microparticles (MP) from platelets and circulating monocytes. These microparticles are rich in TF, so they support FXa generation and, in turn, promote thrombin generation and platelet activation (activated platelets can adhere to the site of injury) [23]. Concurrently, activated platelets release inorganic polyphosphate (polyP) and platelet phospholipids. These substances strongly amplify several steps of the initiation phase of blood coagulation. Specifically, they promote the activation of factors XII, V, and XI and inhibit fibrinolysis and clot lysis [24]. While the initiation phase of coagulation occurs on TF-expressing surfaces, the propagation phase occurs far away, on surfaces that contain procoagulant phospholipids such as activated platelets. A greater activation of FIX by FXIa, and subsequently of FX by the FIXa/FVIIIa complex, occurs on the phosphatidyl serine (PS)-rich platelet surface [24]. On the platelet-activated surface, FXa binds with its cofactor (FVa) and its prothrombin substrate, generating a burst of thrombin [23] or a sufficient amount of thrombin to massively form fibrin fibres [24]. This represents the propagation phase of coagulation, and this process’s cycle to produce a large amount of thrombin that finally cleaves circulating fibrinogen to fibrin. Initially, the fibrin formed is unstable, but subsequently it is stabilised and becomes insoluble (fibrin mesh) under the action of the activated factor XIII (FXIIIa), which is activated by thrombin (FIIa) [23].

Some specific medical conditions, such as prolonged immobilisation during hospitalisation, paralysis or extended travel, atrial fibrillation or severe bradycardia, orthopaedic surgery, COVID-19, cancer, obesity, etc., are associated with venous stasis, which is responsible for the accumulation and activation of procoagulant factors (e.g., thrombin) and the development of VT [31].

## 3. Classification of ACs

Given the multiple factors involved in the coagulation cascade, the ACs may block it at different levels. Therefore, considering their primary mechanism of action, ACs could be classified into three major groups (Table 1): (i) indirect inhibitors of clotting factors IIa and Xa; (ii) direct inhibitors of clotting factors IIa and/or Xa: parenteral and oral direct inhibitors of clotting factor IIa; (iii) inhibitors of the hepatic synthesis of clotting factors dependent on vitamin K (vitamin K antagonists—VKAs).

An important factor in deciding on treatment is the drug’s target. Targeting factor Xa inhibits thrombin activation. The process starts downstream, requires lower AC doses, and has minimal adverse effects due to inhibition of factor Xa [41]. Moreover, the selectivity for factors Xa or IIa is evaluated by the anti-Xa/anti-IIa ratio. UHF inhibits both factors (Xa and IIa) to the same extent (anti-Xa/anti-II ratio = 1). Still, LMWH and ULMWH preferentially inhibit factor Xa proportional to the reduction of the polysaccharide chain. Thus, the anti-Xa/anti-IIa ratio is 2.5 for dalteparin, 3.9 for enoxaparin, and 9.7 for bemiparin (Table 1). Moreover, fondaparinux, the heparin pentasaccharide sequence responsible for ACs interacting with ATIII, inhibits factor Xa (anti-Xa/anti-IIa ratio = ∞) exclusively [40].

The anticoagulant effect and implicit selection of appropriate ACs depend on pharmacokinetic properties. Generally, their bioavailability is not influenced by food intake, but there are some exceptions: increasing the bioavailability of rivaroxaban (≥15 mg/day) and decreasing the bioavailability of betrixaban. The initiation and monitoring of the AC treatment should consider the elimination route (renal or biliary) and its biotransformation. Most ACs are metabolized under cytochrome P450 (CYP) isoenzymes (e.g., CYP3A4, CYP2C9, CYP3A5, CYP1A2, etc.). Therefore, their pharmacokinetic properties should be considered for establishing the benefit/risk balance (Table 2) [42].

From a clinical perspective, the pharmacokinetic profiles of each anticoagulant vary in their implications for drug choice, dosage, and safety. Their pharmacokinetics depend on the route of administration and the molecular structure. UFH is characterised by a rapid onset of action when administered via the i.v. route, it exhibits extensive binding to nonspecific plasma proteins and clearance via the reticuloendothelial system [43,44,45,46]. LMWHs and fondaparinux are administered s.c. and exhibit a predictable bioavailability. Compared with UFH, these drugs have longer half-lives, improved subcutaneous bioavailability, and a more predictable dose–response relationship for anticoagulation, allowing for once- or twice-daily administration (for LMWHs). While dose adjustment is based on the patient’s weight, specific clinical conditions (renal impairment, pregnancy, obesity, etc.) require careful consideration due to potential alterations in pharmacokinetics [7,27,46,47,48,49,50,51,52,53,54]. Compared to parenteral drugs, which have a rapid onset of action, oral drugs show more complex absorption and metabolism. VKAs are well absorbed via the oral route and present a high bioavailability (100%). Due to hepatic metabolism and genetic polymorphisms, they show considerable interindividual variability and a delayed onset of action [55,56,57,58]. By comparison, DOACs demonstrate more predictable pharmacokinetics, with rapid absorption and fixed dosing regimens. Dabigatran is predominantly renally eliminated, whereas factor Xa inhibitors such as rivaroxaban, apixaban, and edoxaban are excreted in the urine and metabolised in the liver. Both VKAs and DOACs (except dabigatran) are metabolised by cytochrome P450 enzymes and are susceptible to many drug–drug interactions (DDIs) [42,46,55,56,57,58,59,60].

**Table 2:** The main pharmacokinetic properties of anticoagulant drugs.

Drug Class	Drugs	Bioavailability	Half-Life	Plasma Protein Binding	CYP Metabolism (Enzymatic Substrate)	Elimination (Primary Route)	Posology and Route of Administration (Most Frequent Administration)	References
UFH	Heparin	100% (i.v.), variable (s.c.)	0.5–1.5 h	>90%	-	reticuloendothelial system, R	Prophylaxis: maximum 15,000 IU BID or TID divided into 2–3 doses/day (prophylaxis) Treatment: i.v. bolus maximum 10,000 IU (50–100 IU/kg) ± maximum 400–600 IU/kg daily in continuous infusion or by 2–4 h i.v. injections * or maximum 250 IU/kg/dose (s.c.) BID	[43,44,45,46]
LMWHs	Dalteparin Enoxaparin Nadroparin Reviparin Tinzaparin	>90%	1.5–6 h	<UFH	-	R	** dalteparin (s.c., i.v.) 2500–5000 IU QD or BID enoxaparin (s.c., i.v.) 20 mg–200 mg/day (divided in 1 or 2 doses) nadroparin (s.c., i.v.) 1900–8550 IU anti-Xa, OD or BID reviparin (s.c., i.v.) 143 IU anti-Xa/kg divided in 2 doses (maximum 10,307 IU/day) tinzaparin (s.c., i.v.) 175 UI anti-Xa/kg (3500–18,000 anti-Xa units QD)	[7,46,47,48,49,50,51,52,53]
ULMWHs	Bemiparin	96%	5.3 h	N/A	-	R	s.c. 2500–25,000 IU QD i.v. bolus: 8 units/kg/hr	[43,44,61,62]
Indirect inhibitors of clotting factor Xa (parenteral)	Fondaparinux	>97%	17–21 h	94% ***		R	2.5 mg OD (s.c., i.v.)	[27,46,54]
Heparinoids	Danaparoid	100%	25 h	N/A	-	R	750 anti-factor Xa units BID (s.c.) i.v. bolus: 1250–3750 anti-factor Xa units + i.v. infusion in decreased doses starting at 400 units/h	[37,63,64]
	Pentosan polysulfate	1%	24–34 h	N/A	-	R (6%)	p.o. 100 mg TID administered on an empty stomach	[27,65]
Parenteral direct inhibitors of clotting factor IIa	Argatroban	100%	39–51 min	54–55%	CYP3A4/5	Faeces (65%), R (22%)	2 mcg/kg/min in continuous i.v. infusion i.v. bolus: 350 mcg/kg starting and maintenance: 15–30 mcg/kg/min (5–6.5 min)	[27,66]
	Desirudin	100%	2 h	****	-	R (40–50%)	s.c. 15 mg BID	[27,67]
	Lepirudin	100%	1.3 h	3%	-	R	i.v. bolus: 0.4 mg/kg, then 0.15 mg/kg/h in continuous infusion	[27,68]
	Bivalirudin	100%	25 min	*****	-	R (20%)	i.v. bolus: 0.75 mg/kg + 0.25–1.75 mg/kg/h in i.v. infusion	[27,69]
Direct oral inhibitors of clotting factor IIa	Dabigatran	3–10	12–17 h	35%	-	Biliary excretion (20%), R (80%)	prophylaxis: 110 mg QD p.o. (starting dose) 220 mg QD p.o. (maintenance dose)	[46,70]
Direct oral inhibitors of clotting factor Xa	Rivaroxaban	>80% (↑ by food, if dose ≥ 15 mg)	5–13 h	>92%	CYP3A4/5, CYP2J2	R (66%), Faeces (28%)	2.5 mg BID p.o. + acetylsalicylic acid, clopidogrel, ticlopidine Day 1–21: 15 mg BID p.o. (VTE treatment, secondary prophylaxis); Day 22–180: 20 mg QD p.o. Day 181⟶: 10 or 20 mg QD p.o. >15 mg, tablets should be administered with food	[42,46,59]
	Apixaban	50%	9–14 h	>92%	CYP3A4/5, CYP1A2, CYP2J2, CYP2C8/9/19	Faeces (<60%), R (~25%)	2.5–5 mg BID p.o.	[46,60]
	Edoxaban	62%	10–14 h	~55%	CYP3A4/5	R (50%), Biliary excretion (50%)	60 mg QD p.o.	[46,71]
	Betrixaban	34% (↓ by food)	19–27 h	60%	CYP1A1 CYP1A2 CYP2B6 CYP2C9 CYP2C19 CYP2D6 CYP3A4	Faeces (85%), R (11%)	160 mg QD p.o. (starting dose) 80 mg QD p.o. (maintenance dose)	[42,72]
VKAs	Warfarin	100%	36–42 h	99%	CYP2C9/8/19 CYP3A4 CYP1A2	R (80%), Faeces (20%)	2–15 mg QD p.o.	[55,56,57,58]
	Acenocumarol	60%	8–11 h	>98%	CYP2C9	R	2–10 mg QD p.o.	[55,56,57,73,74,75]
	Phenprocumon	~100%	160 h	99%	CYP2C9 CYP2C8 CYP3A4	R	0.75–9.0 mg QD p.o.	[55,56,57,74]

BID (bis in die)—twice a day; CYP—cytochrome P450; i.v.—intravenous; IU—Internation Unit; LMWHs—low-molecular-weight heparins; N/A—not available information; QD (quaque die)—once a day; p.o.—per os; R—renal elimination; s.c.—subcutaneous; TID (ter in die)—three times a day; UFH—unfractionated heparin; ULMWHs—ultra-low-molecular-weight heparins; VKAs—vitamin K antagonists; VTE—venous thromboembolism. * Heparin doses are established based on the activated partial thromboplastin time (aPTT) value and the patient’s diagnosis and body weight; ** LMWH posology is established according to the heparin type, patients’ condition, and risk; *** Fondaparinux does not bind significantly to other plasma proteins except ATIII; **** Desirudin binds specifically and directly to thrombin (IIa); ***** Bivalirudin does not bind to other plasma proteins except thrombin (IIa) and red blood cells; ↑—increased; ↓—decreased.

Patient-specific characteristics such as advanced age, impaired renal or hepatic function, comorbidities, and polypharmacy significantly influence the pharmacokinetics and safety profile of oral anticoagulants. Variations in half-life and clearance can increase the bleeding risk and often require treatment adjustment, or even discontinuation. Anticoagulant agents should be used cautiously or avoided in cases of severely reduced kidney function due to their primary renal elimination (e.g., LMWHs, fondaparinux, DOACs, VKAs) [57,76,77]. Oral anticoagulants are more frequently associated with clinically relevant DDIs than parenteral agents, mainly due to their complex hepatic metabolism and intestinal transport mechanisms. Consequently, hepatic metabolism is a limiting factor for using DOACs in severe liver impairment. Finally, oral anticoagulants cross the placenta and are teratogenic, leading to adverse foetal effects, and, therefore, they must be avoided during pregnancy, whereas UFH and LMWHs are considered safe options [57,76,77].

### 3.1. Indirect Inhibitors of Clotting Factor IIa and Xa

#### 3.1.1. Unfractionated Heparin (UFH)

Many animal sources can be used to extract UFH, a mucopolysaccharide composed of sulfated D-glucosamine and D-glucuronic acid (1:1). Heparin is released from human or animal mastocytes (tissue leukocytes). The first used was the bovine lung in the 1930s, followed by the porcine mucosa in the 1950s [78], which is nowadays the most frequently used [79]. UFH is sometimes obtained from bovine mucosa, but studies have shown that the antithrombotic activities of porcine heparin are superior to those of bovine heparin [79]. The length of the heparin chain, along with its sulfation pattern, greatly influences heparin’s biological activity.

**Figure 3:**
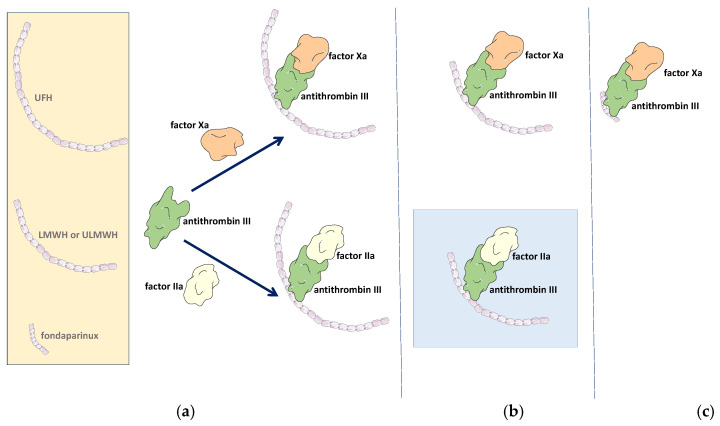
Indirect inhibition of clotting factor IIa and Xa, via ATIII: (**a**) complexes of UFH-antithrombin III and factor IIa and/or Xa; (**b**) complexes of LMWH/ULMWH-antithrombin III and factor Xa and/or IIa (the activity of the complex with factor IIa from the blue frame is very low); (**c**) fondaparinux-antithrombin III-factor Xa complex [81,82]. Graphical representation of drugs (the length of the chain) is proportional to molecular weight.

The main pharmacological effect of heparin is represented by the anticoagulant activity that results after its binding to ATIII. As a result, it enhances the interaction between ATIII and thrombin (clotting factor IIa) by forming a ternary complex UFH–ATIII–thrombin, thereby inhibiting thrombotic activity through an indirect mechanism, thus presenting an indirect antithrombotic mechanism [37]. In the same way, inhibition of factor Xa occurs via the UFH-ATIII complex. It requires a pentasaccharide sequence for factor Xa binding, whereas the formation of the UFH–ATIII–thrombin ternary complex requires the presence of at least 18 saccharide units, including the pentasaccharide sequence [5,80] (Figure 3a).

Additional properties of heparin may influence its binding to other proteins or cells, which may lead to (i) inhibition of its anticoagulant effects mediated by nonspecific binding to plasma proteins (e.g., histidine-rich glycoprotein, fibrinogen, apolipoprotein B, and fibronectin); (ii) heparin-induced thrombocytopenia (HIT) determined by platelet activation and interaction of IgG antibodies with heparin-platelet factor 4; (iii) osteopenia and osteoporosis determined by heparin binding to osteoblasts, inhibition of the bone-forming osteoblasts, and stimulation of bone-resorbing osteoclasts; (iv) antiproliferative activity by inhibiting the processes of malignant cells growing and angiogenesis (e.g., selectins, heparinase, cytokines, vascular growth factors); (v) anti-inflammatory activity due to binding of inflammatory mediators and enzymes (e.g., cytokines, chemokines, etc.) [20,37,83,84].

Furthermore, UFH presents a very high variability in the dose–response relationship. In this context, treatment monitoring is mandatory [85]. UFH indications are prophylaxis and treatment of venous thromboembolism (VTE), prophylaxis of the thrombotic stroke, peripheral arterial embolism, ischemic complications (unstable angina, non-ST-elevation myocardial infarction—NSTEMI), and prophylaxis in cardiac surgery or hemodialysis [38]. In addition to its use as an anticoagulant, some studies investigated the potential therapeutic role of heparin in allergic pathologies (e.g., asthma, rhinitis), reproductive, cardiovascular or renal diseases, metastases, viral or protozoal infections, etc. [20,62,84].

UFH has some advantages compared to other AC classes, such as rapid onset, very long experience in clinical use, low cost, and rapid reversal of adverse effects after sulfate of protamine administration [20]. However, heparin-induced thrombocytopenia (HIT) and bleeding risks, i.v. administration, and frequent monitoring could limit its use [86].

#### 3.1.2. Low and Ultra-Low-Molecular-Weight Heparins

The LMWHs and ULMWHs are obtained through chemical depolymerisation of UFH, such as deaminative cleavage using nitrous acid (nadroparin, dalteparin, reviparin) or isoamyl nitrite (certoparin), alkaline depolymerisation after the addition of benzoyl groups (enoxaparin), peroxidative cleavage (ardeparin, parnaparin), or enzymatic depolymerisation of UFH using heparinase I (tinzaparin) [78,87]. Reducing the molecular weight of the polymeric chain improves the properties of LMWHs and ULMWHs compared to UFH. Thus, the LMWHs’ binding to other proteins (except ATIII) is reduced, and implicitly, the risk of LMWHs’ adverse reactions (e.g., bleeding, HIT, bone loss, etc.) is reduced, too. Also, LMWHs have an improved dose–response effect, a longer half-time, and a better ratio of anti-factor Xa activity relative to anti-factor IIa activity compared to UFH [62,88]. More than 50% of LMWHs are not able to form the ternary complex with ATIII and thrombin due to their short chain; thus, their anticoagulant activity occurs after the inhibition of factor Xa, mediated by ATIII (Figure 3b) [80].

A retrospective study shows a decrease in hospital death and reinfarction in patients with acute ST-elevation myocardial infarction (STEMI), treated with enoxaparin versus UFH [38]. Enoxaparin has a broader range of indications approved by the Food & Drug Administration (FDA) [86].

LMWHs possess some advantages over UFH, such as greater efficacy, improved safety, the ability to use fixed doses without monitoring and the possibility of treating patients at home, and greater adherence to treatment due to the reduction in the number of administrations and hospitalisation costs [38]. Therefore, LMWHs replace UFH in many situations, such as prophylaxis and treatment of VTE, prophylaxis of complications of acute coronary syndrome or major surgery, etc., but the slower onset of the action of LMWHs or ULMWHs compared to UFH represents a disadvantage of these drugs [85]. Other disadvantages of LMWHs compared to UFH include renal elimination, which limits their use in patients with renal failure. Additionally, the high cost of treatment and the risk of HIT or bleeding (even if reduced) could limit the use of LMWHs [62,86,88].

ULMWHs (e.g., bemiparin, semuloparin) were obtained after further reduction of the polymer chain from the heparin structure. This increased the efficacy of the new compounds (higher ratio of anti-factor Xa to anti-factor IIa activity) and reduced their risks (e.g., bleeding, HIT, osteoporosis). Bemiparin is effective in a single daily dose [89,90].

Indications of LMWH and ULMWH include the following [10,20,90,91,92,93,94,95,96,97,98]:

- VTE prophylaxis in orthopaedic, bariatric, or general surgery (e.g., nadroparin, enoxaparin, bemiparin, tinzaparin, certoparin, etc.);
- Prophylaxis of clotting in haemodialysis patients (e.g., nadroparin, dalteparin, tinzaparin, etc.);
- Treatment of deep vein thrombosis (DVT), including cancer-associated thrombosis (e.g., certoparin, nadroparin, enoxaparin, bemiparin, etc.) or treatment of pulmonary embolism (PE) (e.g., enoxaparin, dalteparin, etc.);
- Traumatic conditions such as trauma, burns, spinal injury (e.g., enoxaparin, reviparin, etc.);
- Prophylaxis of ischemic complications in acute coronary syndromes (unstable angina, NSTEMI myocardial infarction) (parnaparin, enoxaparin, etc.);
- Bridge therapy (especially enoxaparin, tinzaparin) prior to starting VKAs in AF, heart valve replacement, history of VTE, etc.

The anti-inflammatory properties of enoxaparin are also being investigated. These are determined by the inhibition of NF-kb translocation and activation with the reduction of some pro-inflammatory cytokines (TNFα, IL-8, IL-6, IL-1beta) [99]. The potential antiproliferative effect of dalteparin in ovarian cancer is being investigated [96].

#### 3.1.3. Indirect Inhibitors of Clotting Factor Xa

The design and development of synthetic heparin derivatives were based on the observation that a specific oligosaccharide sequence is responsible for the anticoagulant activity [80,100]. Thus, the synthesis of this essential pentasaccharide sequence led to the development of fondaparinux and other heparin mimetic drugs, which have some advantages, such as fewer adverse reactions due to their simplified structure and smaller molecular size [100,101]. Therefore, fondaparinux is a synthetic drug consisting of the heparin pentasaccharide sequence responsible for interacting with antithrombin III. Accordingly, fondaparinux exerts its anticoagulant activity by forming a ternary complex with ATIII-factor Xa (Figure 3c), with high affinity [80]. It has a benefit-to-risk ratio comparable to LMWH and warfarin. For this reason, it has been approved for the prevention of VTE after major surgery, such as in high-risk orthopaedic patients. Some studies suggest that fondaparinux can be used for the initial treatment of VTE, due to its safety and efficiency compared to heparin or LWMH [102]. Also, it can be used for VTE prophylaxis in patients with myocardial infarction [102]. Because of its high elimination half-life (15–41 h) and dose-independent effect, fondaparinux can be administered subcutaneously (s.c.) daily [102]. A study performed by Senzolo et al. showed the therapeutic potential of fondaparinux in the treatment of portal vein thrombosis in patients with cirrhosis [103].

Referring to other compounds in this class, a study conducted by Harenberg et al. showed that the long elimination half-life of idraparinux (60 days) was responsible for the bleeding complications [104] that determined the withdrawal of idraparinux from clinical trials [105]. Moreover, another study reported unclear evidence of the safety and efficiency of idraparinux or idrabiotaparinux (a derivative of idraparinux) in VTE treatment [103,106].

#### 3.1.4. Heparinoids

Heparinoids are glycosaminoglycan derivatives from heparin (UFH). Heparan sulfates are classified as heparinoids with potential anticoagulant properties due to their binding to ATIII. Differing from UFH, which is presented in mastocytes, heparans are presented on the cell surface or in the extracellular matrix [107]. They are obtained by selective desulfation of heparin methods. Among these, alternative ways of obtaining heparan sulfates are currently under investigation, such as chemical or chemoenzymatic synthesis, sulfation of polysaccharides, and metabolic engineering [108].

Another representative of the class is danaparoid, a mixture of heparan sulfate (84%), dermatan sulfate (12%), and chondroitin sulfate (4%). Danaparoid has low specific activity against factor Xa and is mainly used for HIT treatment. It is approved in the prophylaxis of postoperative DVT in patients undergoing general or orthopaedic surgery and for the treatment of disseminated intravascular coagulation, too. Its main disadvantages are a long half-life (25 h), which limits its use when surgery or an invasive procedure is urgent, and the lack of its antidote [37,106].

### 3.2. Direct Inhibitors of Clotting Factors IIa and Xa

#### 3.2.1. Parenteral Direct Inhibitors of Thrombin (Clotting Factor IIa)

Hirudin, a natural antithrombin substance, represented the starting point for the development of a new class of parenteral anticoagulants that directly inhibits the activity of clotting factor IIa (thrombin). Hirulogs or hirudin analogues (lepirudin, desirudin, bivalirudin) were obtained by genetic engineering, and argatroban was obtained by chemical synthesis. They were approved for the treatment of VT in patients with HIT [27]. Bivalirudin is approved for use in percutaneous coronary interventions (PCIs), as the primary treatment for percutaneous transluminal coronary angioplasty. Moreover, bivalirudin has a low level of immunogenicity [109].

Argatroban is a direct thrombin inhibitor and is recommended as an alternative to intravenous heparin for the treatment of HIT and for anticoagulation during percutaneous coronary intervention (PCI). Due to its low molecular weight, it is effective in the treatment of microvascular disorders [109,110]. Another analogue, desirudin, is recommended in the post-operative prevention of VTE in patients undergoing elective hip replacement surgery [111].

These drugs have some disadvantages, such as risk of anaphylaxis (lepirudin), narrow therapeutic window (lepirudin), immunogenic effect with antibody synthesis (desirudin), and risk of bleeding (argatroban, lepirudin, desirudin) [112].

A retrospective study performed by Bain and Meyer compared the efficacy and safety of the three agents from this pharmacologic class. The results confirmed that bivalirudin has the best profile, because it determined a lower incidence of significant bleeding (7%) compared to argatroban (22%) and lepirudin (56%) [113].

#### 3.2.2. Direct Oral Anticoagulants (DOACs)

Two categories of DOACs were approved on the market in recent years: direct factor IIa inhibitors (dabigatran, melagatran, ximelagatran) and direct factor Xa inhibitors (rivaroxaban, apixaban, edoxaban, and betrixaban). Compared to other ACs, they have some advantages, such as oral administration, rapid onset of action, slight interindividual variation and ability to standardise doses, reduced number of drug interactions, lower risk of intracranial bleeding, and a reduced need for treatment monitoring [85,114]. Ultimately, they can significantly improve patient adherence to the anticoagulant treatment [57].

A meta-analysis by Ruff et al. revealed that, compared with VKAs, DOACs (dabigatran, rivaroxaban, apixaban, and edoxaban) had a lower incidence of major bleeding, including haemorrhagic stroke and intracranial haemorrhage. The same study showed that the incidence of systemic embolic events and all-cause mortality was reduced in DOAC treatment. On the other hand, significantly more ischemic strokes and an increase in gastrointestinal bleeding were observed [115]. Other data suggest that direct factor IIa inhibitors could be administered in the prevention of recurrent clots (in legs or lungs) because there is no difference between the efficacy of these compounds and standard therapy [116]. In addition, apixaban and rivaroxaban are recommended in VTE prophylaxis in patients with nonvalvular atrial fibrillation (AF) or orthopaedic replacement surgery [62,117,118].

However, some particularities that could limit the use of DOACs could be identified:

- DOAC treatment requires periodic assessment of renal function [46,57,114,119];
- Edoxaban is not recommended in patients with high general bleeding risk, and rivaroxaban is not recommended in patients with high gastrointestinal or heavy menstrual bleeding risk [46,57,114,119];
- Dabigatran is not recommended in patients with renal impairment, dyspepsia, or different gastrointestinal diseases that could reduce its absorption (e.g., gastrectomy, short bowel, etc.) [46,57,114,119];
- Dabigatran and edoxaban require bridge therapy in certain situations and should not be crushed for nasogastric tube administration [46,57,114,119];
- DOACs and their antidote costs are very high for clinical use [86];
- The risk of gastrointestinal bleeding is higher in elderly patients treated with DOAC [86];
- Betrixaban is not recommended in DVT and PE treatment, prevention of stroke or non-valvular AF, or for DVT prophylaxis in orthopaedic surgery [119];
- There is some evidence that has associated dabigatran with a higher risk of ischemic stroke [120];
- The prodrug ximelagatran (active form: melagatran) presented a higher risk of hepatic toxicity and was withdrawn from the pharmaceutical market in 2006 [114,121].

According to the ARISTOTLE trial, the apixaban incidence of adverse events is similar to that of warfarin [122]. Other studies, such as RE-LY (dabigatran 150 mg) [122], ROCKET (rivaroxaban 20 mg or 15 mg in patients with CrCl 30–49 mL/min) [123], and ENGAGE-AF-TIMI (edoxaban 60 mg) [124] demonstrated a higher incidence of gastrointestinal bleeding in patients treated with DOACs compared with those treated with warfarin [122,123,124].

### 3.3. Vitamin K Antagonists

Hepatic synthesis of some clotting factors (II, VII, IX, X) and clotting regulatory factors (proteins C and S) depends on the carboxylation of glutamic acid into gamma-carboxyglutamic acid from different proteins (active clotting factors) [125]. This process requires the presence of a γ-glutamyl carboxylase and is dependent on the reduced form of Vitamin K [73]. VKAs inhibit vitamin K oxide reductase (VKOR1), an enzyme essential for the reduction of vitamin K 2,3-epoxide to the active form (vitamin K hydroquinone) [126]. Thus, VKAs lead to (i) depletion of the reduced form of vitamin K; (ii) a decrease in some coagulation factor hepatic synthesis (the active forms of some coagulation factors); and (iii) inhibition of the carboxylation of some regulatory anticoagulant proteins (proteins C, S, and Z) [125]. In the absence of vitamin K, the liver synthesises some special proteins, PIVKA (proteins induced by vitamin K absence), devoid of pharmacological effect and used as biomarkers of anticoagulant overdose [127].

VKAs therapy is mainly recommended in the following cases:

- Patients with AF (for the primary prevention of stroke);
- Patients with DVT and PE (for the prevention of recurrent thromboembolism);
- Patients with valvular heart disease or prosthetic cardiac valves (for the prevention of thrombosis) [128].

Due to their pharmacokinetic properties, VKAs have more interactions (including with food) and a slow onset and offset. Thus, initiation of anticoagulant therapy with adequate doses of a VKA requires an overlap period with heparin of at least 5 days. Moreover, the therapeutic range of VKA is narrow. During VKA treatment, the International Normalised Ratio (INR) should be maintained between 2.0 and 3.0, especially during the initiation period [128].

Because of all these aspects, adherence to VKA treatment could be worsened [57]. According to the results of different studies, above 40% of the time, the INR value in patients treated with VKAs is outside of the above-mentioned range [57]. However, a primary advantage of VKAs is represented by their possibility to be used in patients with severe renal failure [57].

## 4. The Biological Effects of Anticoagulants

### 4.1. Antithrombotic Activity

#### 4.1.1. Deep Vein Thrombosis

Most commonly, thrombus formation in the deep veins of the limbs, pelvis, or arms is driven by activation of the coagulation cascade and can lead to DVT. Also, the thrombus can be formed in the superficial veins of the lower limbs. The main clinical manifestations of DVT are represented by unilateral leg pain, redness, and swelling [129]. Subsequently, through dislocation and migration, the thrombus can remain blocked in the lungs and cause PE.

Anticoagulant treatment is helpful in controlling symptoms, preventing thrombus formation and progression, and reducing the risk of post-thrombotic syndrome and PE. The main anticoagulation strategy in DVT consists of the following:

(i) Parenteral treatment (UFH, LMWH, fondaparinux) overlapped with oral VKA, followed by oral therapy with VKA;
(ii) Parenteral treatment (UFH, LMVH, fondaparinux) followed by oral therapy with dabigatran (RECOVER, RECOVER II trials) or edoxaban (HOCUSAI trial);
(iii) Oral therapy with apixaban (AMPLIFY trial) or rivaroxaban (EINSTEIN trial) [130,131,132].

Regarding the recurrence of VTE, sulodexide, a combination of heparan sulfate (80%) and dermatan sulfate (20%) [133], used after discontinuation of anticoagulant therapy, has been shown to reduce the recurrence of VTE [134].

#### 4.1.2. Pulmonary Embolism

PE is represented by the dislocation of clots or clots’ fragments from the vein walls and their movement to the pulmonary arteries through the heart [135]. This produces occlusion of the pulmonary arteries, leading to poor perfusion of the alveolar capillary bed and then to an imbalance between ventilation and perfusion. Therefore, respiratory failure will occur. Thus, the most serious complication of DVT is PE [136]. PE is associated with chest pain, shortness of breath, tachypnoea, and tachycardia [135,137].

Initiation of treatment in patients with PE is recommended to be realised with UFH (t_max_ is the lowest), administered in bolus, because ACs with increased half-life are contraindicated in hemodynamically unstable patients [46]. Despite UFH and LMWH being the cornerstone of anticoagulant therapy, subcutaneous fondaparinux has emerged as a safe and effective alternative. Initial parenteral anticoagulation is not necessary for rivaroxaban or apixaban but is required for dabigatran or edoxaban. Moreover, the overlap of parenteral anticoagulation with VKAs is requested, too [138].

#### 4.1.3. Orthopaedic Surgery

(a) In patients with major orthopaedic surgeries, particularly of the lower limbs or pelvis, there is an increased risk of venous thrombosis (coagulation activation), on the one hand due to the long period of patient immobilisation associated with venous stasis, and on the other hand due to the release of tissue factor (TF) from the bone during surgery. Thus, the following recommendations should be taken into consideration in orthopaedic surgery [130]: LMWH, apixaban, and rivaroxaban are used in case of knee or hip surgeries, and fondaparinux, UFH, and warfarin could be used as second-line therapeutic options when the others are contraindicated or cannot be administered.
(b) LMWH, UFH, and fondaparinux are used for DVT prophylaxis in patients with hip fracture surgery.

#### 4.1.4. Cerebrovascular Diseases

Cerebrovascular disease is a main cause of death and disabilities associated with the disruption of cerebral blood flow and the deprivation of brain cells of oxygen and nutrients. Cardiovascular diseases (hypertension, AF, etc.), and diabetes mellitus are the main contributors to cerebrovascular disease [139,140]. AF increased the risk of stroke above fivefold [57]. Therefore, prevention of cardioembolic ischemic stroke associated with AF requires the use of oral anticoagulants. The studies showed that the risk of stroke or systemic thromboembolism and all-cause mortality was reduced in the group treated with oral anticoagulants in comparison to the placebo group [139,140]. It was observed that the rate of stroke is reduced by 60% in patients with AF treated with VKAs [141]. Thus, warfarin is efficient and recommended in the treatment of patients with AF and ischemic stroke or transient ischemic attack. Superior results were obtained with apixaban, rivaroxaban, edoxaban, or dabigatran (including in patients over 75 years of age with higher doses or in severe renal failure with reduced doses) [139,142]. An important problem related to cerebrovascular disease is represented by the reinstitution of therapy after an intracranial haemorrhage episode. It was noticed that the reinstitution of anticoagulation was associated with a lower risk of thromboembolic complications [143].

#### 4.1.5. Anticoagulants Used in Heparin-Induced Thrombocytopenia

A rare prothrombotic disorder produced by the administration of UFH or LMWH is represented by HIT. Abnormal antibodies responsible for platelet activation are generated after heparin administration [144,145]. VKAs are contraindicated in patients with HIT and platelet count under 150 × 10^9^/L [146]. Initially, parenteral non-heparin anticoagulants were used in the treatment of HIT [144,145]. Thus, danaparoid and argatroban could be used in patients with HIT without renal impairment. According to some studies, bivalirudin could be an alternative in case of urgent cardiac surgery in patients with acute or subacute HIT [88]. Other studies have shown similar efficacy and safety of fondaparinux to parenteral non-heparin ACs in patients with suspected HIT [144,147,148]. A recent study noticed the potential and safe use of DOACs (rivaroxaban or apixaban) in the acute phase of HIT [149]. Administration of dabigatran in HIT was investigated, too, and similar results were obtained [145]. Moreover, no major bleeding or thrombosis was observed in the mentioned studies during the follow-up period [145,149].

#### 4.1.6. Anticoagulants in Antiphospholipid Syndrome

Antiphospholipid syndrome or Hughes syndrome (APS) is another autoimmune hypercoagulable disorder that produces abnormal antiphospholipid antibodies with a high risk of arterial and venous thrombosis, pregnancy-related complications, and other symptoms like low platelets, kidney disease, heart disease, and rash. Antiphospholipid antibodies react against proteins that bind to anionic phospholipids on cell membranes. Anticardiolipin antibodies, β2-glycoprotein 1, and lupus anticoagulant are antiphospholipid antibodies that are thought to clinically cause disease. APS often requires treatment with anticoagulant medication to reduce the risk of recurrent episodes of DVT and improve pregnancy outcomes. Thus, lower aspirin doses are recommended for primary prophylaxis of APS in high-risk patients, and VKAs are recommended for patients with APS and a first episode of VTE. In pregnant women, UFH and LMWH can be administered prophylactically. DOACs are an alternative to VKAs when the latter are contraindicated, or INR is not in the 2–3 range [146].

A few people present APS thrombotic recurrence despite adequate doses of anticoagulant. Therapeutic strategies for anticoagulant-refractory patients include (i) increasing VKA doses; (ii) administration of low-molecular-weight heparin or fondaparinux in addition to antiplatelet therapy; (iii) association of adjunctive treatment with hydroxychloroquine, statins, and vitamin D to anticoagulant therapy [150].

#### 4.1.7. Anticoagulant Therapy in Cancer

Cancer-associated venous thromboembolism is primarily driven by tumour-related tissue factor expression, endothelial injury, and venous stasis. Other pathological factors, such as antiphospholipid syndrome, factor V Leiden mutation, etc., increase the incidence of VTE events in cancer patients. Some therapeutic conditions, such as chemotherapy, surgical interventions, and prolonged immobilisation, are other risk factors. Thromboprophylaxis should be considered for all hospitalised patients with cancer (except those with contraindicated anticoagulation), or before major cancer surgery, or for ambulatory treatment of patients at high risk of VTE, particularly multiple myeloma treated with thalidomide or lenalidomide in combination with chemotherapy or high-dose dexamethasone. Different studies have shown the efficacy and safety of thromboprophylaxis or anticoagulant treatment with enoxaparin, nadroparin, tinzaparin, dalteparin, and bemiparin in patients with various types of cancer [62,151]. A recent study shows a similar efficacy of DOAC with LMWH over a 6-month follow-up for preventing recurrent VTE among patients with cancer and VTE [152].

#### 4.1.8. The Use of Anticoagulants in COVID-19

SARS-CoV-2 infection is associated with a hypercoagulable state, characterised by pulmonary microthrombosis and generalised microthrombosis, leading to a high risk of multiple organ failure. In severe forms of COVID-19, several coagulation markers are increased (D-dimer, factor VIII, fibrinogen, C-reactive protein), while antithrombin III and protein S levels are lower than normal. Very low levels of antithrombin III are characteristic of severe pathologies such as sepsis, disseminated intravascular coagulation, and severe infections, including COVID-19, and are considered a predictive factor of mortality [153,154]. Different studies have shown that markedly elevated D-dimer levels are associated with high mortality in COVID-19 patients [155]. Thrombotic complications, particularly PE, are frequent in COVID-19 patients [156]. Anticoagulant therapy has been associated with reduced mortality in patients with multi-organ failure and sepsis. In this context, LMWHs have been linked to improved outcomes, including the protection of critically ill patients against VTE. Moreover, the anti-inflammatory properties of LMWH may benefit patients with COVID-19, a condition characterised by markedly elevated pro-inflammatory cytokine levels. Current guidelines recommend LMWH use as prophylaxis in all patients hospitalised with COVID-19, in the absence of contraindications [155]. The findings from a study performed by Nab et al. were suggestive of a potential reduction in the risk of in-hospital mortality with a higher dose of LMWH as thromboprophylaxis [156].

The Chest Guideline and Expert Panel Report suggested thromboprophylaxis with LMWHs and fondaparinux over the UHF, and all three categories were recommended for thromboprophylaxis over DOACs [157]. UFH is recommended for patients with severe or terminal chronic kidney disease or with renal replacement therapy, and fondaparinux is recommended in patients with a history of HIT [158]. The use of DOACs in hospitalised patients with COVID-19 was limited by their pharmacodynamic and pharmacokinetic properties, particularly their significant drug interactions (e.g., antivirals, antibiotics, antihypertensives, bronchodilators, and immunosuppressive drugs) [159,160]. In addition to metabolic alterations or acute kidney injury, DOACs can cause an unpredictable and unstable anticoagulant effect, exposing patients to the risk of bleeding or thrombotic complications [159]. However, COVID-19 inpatients can be switched from parenteral ACs (LMWH or UFH) to oral anticoagulant therapy with a DOAC or warfarin upon hospital discharge, for a minimum of 3 months [160,161].

#### 4.1.9. Particular Situations of Anticoagulant Use

##### Renal Impairment

Chronic kidney disease (CKD) has a high risk of thrombotic events; anticoagulation in these patients is very important. UFH and LMWH are considered safe for non-dialysis patients. In the first three stages of CKD, the preferred medication is represented by DOACs, and in the end-stage, warfarin is considered the first-line therapy. The choice of treatment in the fourth stage of CKD should take into account the drug’s pharmacokinetics (DOACs or warfarin) and the patients’ condition [162].

Some evidence has shown a better risk–benefit profile of DOACs compared to VKAs, for the prevention of thromboembolic events in patients with nonvalvular AF [163]. A meta-analysis suggested that, in patients with AF and CKD, DOACs should have an improved effect than warfarin in thromboembolism prophylaxis; apixaban seems to be superior to other DOACs [164]. But, in patients with severe renal impairment, the safety and effectiveness of apixaban is similar to warfarin [165]. In dialysis patients with CKD, thromboembolic prophylaxis could be safely and efficiently obtained with UFH and LMWH, drugs that can be used in bridging anticoagulation [94,166]. The literature has suggested that the use of fondaparinux in renal impairment is not recommended [167].

##### Liver Failure

In patients with liver disease, a significant incidence of bleeding or thrombotic events was observed. [168,169]. Even though patients with chronic liver disease may have low plasma levels of coagulation factors, anticoagulant treatment may still be necessary for other complications associated with liver dysfunction. However, the efficacy of LMWH and dabigatran varied with the severity of hepatic failure. Rivaroxaban is less efficient in patients with compensated cirrhosis, and its doses should be increased in order to improve its efficacy [168]. According to the American Society of Gastroenterology, heparin infusion is recommended for the treatment of hepatic VT (portal and mesenteric vein thrombosis). LMWH, VKAs, and DOACs improve portal vein repermeability and are considered safe and effective in patients with cirrhosis. In patients with cirrhosis, administration of enoxaparin reduced the incidence of portal vein thrombosis [170,171].

##### Elderly Patients

The decision to use an anticoagulant drug should take into consideration the patient’s thrombotic risk and major bleeding events produced by the anticoagulant treatment. In this regard, the recommendation for anticoagulants used in elderly patients with AF or VTE is represented by DOACs [172,173]. Moreover, the risk of intracranial haemorrhage in elderly patients was lower during the thromboprophylaxis with DOACs compared to warfarin [174]. For the same drugs, other studies showed similar results regarding the risks of bleeding or stroke in elderly patients with AF (≥75 years) [175,176].

##### Pregnancy and Lactation

LMWHs, fondaparinux, and apixaban are included in pregnancy category B (no risk in animal studies); UFH, dabigatran, rivaroxaban, and edoxaban are included in category C (risk cannot be ruled out, but the potential benefits of the drug may outweigh the risks); and warfarin is included in category D (evidence of risk to the foetus). On the other hand, VKAs, UFH, and LMWHs are not present in breast milk. VKAs and apixaban cross the placenta, and probably other DOACs as well. Despite VKAs having a dose-dependent foetal risk (foetal loss, embryopathy, bleeding), they can be administered in the immediate postpartum period [177,178].

Even if UFH does not cross the placenta due to its high molecular weight, the presence of benzyl alcohol as a bacteriostatic preservative in pharmaceutical formulations can induce a neurological syndrome in neonates. UFH is used in the third trimester and has an increased risk of antenatal bleeding [177].

LMWHs present a higher bioavailability compared to UFH, but require the determination of the anti-Xa level 4–6 h after administration [178]. Administered during pregnancy, LMWHs and fondaparinux do not increase foetal or neonatal risks [177]. LMWH, UFH, and warfarin could be used for preventing thrombosis in pregnant women with mechanical heart valves, while DOACs are contraindicated and argatroban should be used with caution during pregnancy [178].

### 4.2. Anti-Inflammatory Effect

Antithrombin III, an endogenous anticoagulant activated by some clotting factors (IIa), induces the release of endothelial PGI2 after its binding to heparan-sulfate proteoglycans. As a result of this process, ATIII inhibits platelet activation and suppresses leukocyte capture and rolling along the vessel wall. Furthermore, ATIII reduces the inflammatory response by binding to specific receptors (LRP-1, CD-13, etc.) and by reducing the IL-6, TNF-α levels, and NF-κB activity; although, the ATIII level is decreased in different pro-inflammatory conditions [153]. Moreover, some studies showed the anti-inflammatory potential of heparin and LMWH. Accordingly, it was observed that heparins negatively influence the production of pro-inflammatory cytokines (TNF-α, IL-8, IL-6, etc.) by inhibiting the activation of NF-kB, an essential transcriptional factor that mediates inflammatory responses [99,179].

### 4.3. Anticoagulant in Wound Healing

The wound-healing process involves haemostasis, local inflammation, cell proliferation, and tissue remodelling. In this situation, interactions occur between extracellular matrix constituents and neutrophils, macrophages, fibroblasts, epithelial or endothelial cells, etc. Cytokines, growth factors, and proteolytic enzymes are involved in the healing process [180].

In vivo and in vitro studies have demonstrated the favourable role of heparin in wound healing by increasing fibrinolytic activity and capillary circulation whilst reducing oedema due to its anti-inflammatory effect. Therefore, heparin repairs the endothelial cell and increases the speed of tissue repair [181]. A clinical study regarding the use of heparin irrigation showed a tendency for better wound healing of superficial burns (1st and 2nd degree) [182].

Many drug delivery systems containing heparin were formulated and tested for accelerating wound healing. For example, some experimental or clinical studies have demonstrated the beneficial effects of heparin-based formulations (e.g., hydrogel dressings encapsulated with heparin and basic fibroblast growth factor, ibuprofen-loaded heparin-modified thermosensitive hydrogel, aqueous heparin solution spray aseptically on burn wounds, polypeptide/heparin composite hydrogels, etc.) [183,184,185,186]. After 10 days of administration, enoxaparin induced better wound healing (as assessed by histological examination) than UFH in an experimental model [187]. In another study on diabetic wound healing, a chitosan hydrogel loaded with bemiparin developed high-quality new dermal tissue [188]. A narrative review performed by Solak et al. presented the possible use of LMWHs, especially enoxaparin, in dermatological disorders such as lichen planus, recurrent aphthous stomatitis, chronic urticaria, and contact hypersensitivity [189].

Nevertheless, some studies have shown no improvement in wound healing and even the presence of complications. For example, a recent experimental study performed on female rats showed no significant difference between the anticoagulants used (enoxaparin, dabigatran, or rivaroxaban) and the control group (physiological saline solution), in terms of histological score or clinical lesions [190]. Moreover, the use of warfarin during the perioperative period in facial plastic and reconstructive surgery leads to an increased risk of postoperative infections and perioperative bleeding [191].

### 4.4. Anti-Viral Activity

UFH and LMWH are biomolecules with a high number of negative charges. According to some studies, they can interact with positively charged regions of glycoproteins on the cell surface and thereby prevent viral binding to these regions. Thus, these electrostatic interactions could prevent viral infection (e.g., hepatitis C, herpesvirus) [96,192].

### 4.5. Heparin Used as Drug–Polymer Conjugate

Recent studies present the potential of heparin in antineoplastic–prodrug therapy. The polymer–drug conjugate offers advantages, including low immunogenicity and toxicity, increased bioavailability, improved drug solubility, and controlled drug release. [193]. For example, an UFH conjugate with folic acid and paclitaxel had cytotoxic activity against MDA-MB-231 human breast cancer cells [194]. A complex with chlorambucil and cysteine was tested on HaCaT normal cells and HeLa cervical cancer cells [195]. The antiproliferative effects of a complex with 5-fluorouracil and cisplatin were observed on NCI-H460 lung cancer cells [196]. Doxorubicin was conjugated with UFH or dalteparin, and the complexes formed were tested for antitumoral activity [197].

## 5. Anticoagulants’ Main Risks

Anticoagulants have proven their efficacy; however, several risks accompany their therapeutic success. Repercussions of anticoagulant therapy may affect not only the coagulation process but also involve other systems (e.g., the immune and bone systems).

### 5.1. Haemorrhagic Risk

Haemorrhage is one of the common long-term risks associated with anticoagulant treatment [198]. Gastrointestinal and intracranial haemorrhages are among the most reported complications of anticoagulants. Although several anticoagulant classes are available, gastrointestinal bleeding risk has been found to be similar for oral ACs, warfarin, and LMWHs [199]. In comparison, a low risk of gastrointestinal bleeding was reported for DOACs [200], but the risk of intracranial haemorrhage may increase up to 10-fold during long-term DOAC therapy [201]. Thus, various scoring systems have been developed to assess the bleeding risk during anticoagulant therapy [202,203]. The research group led by Wolfe determined that among DOACs, the risk of intracranial haemorrhage was lowest for dabigatran and highest for rivaroxaban [204]. These results are sustained by the findings of Wu and collaborators, who concluded that, compared to aspirin, dabigatran was similar, but rivaroxaban had a higher risk of intracranial haemorrhage [205]. Mild bleeding may require only temporary discontinuation of anticoagulation, whereas severe bleeding necessitates the use of specific reversal agents [206,207].

### 5.2. Heparin-Induced Thrombocytopenia

HIT is a life-threatening event that affects a small number of patients (up to 1%), often under UFH therapy [208]. It is classified into two types: type I (a non-immunologic reaction between heparin and circulating platelets) and type II (a hypersensitivity reaction, immunoglobulin (Ig) G antibody-mediated) [209,210]. Type II triggers hypercoagulability induced by platelet-activating anti-PF4 antibodies [211]. Highly specific, sensitive laboratory tests were developed for the diagnosis [212] of this ailment, which occurs within days or weeks of initiation of heparin treatment [209]. Fondaparinux and DOACs can be used as alternatives to heparin in HIT [213]. Argatroban and bivalirudin were successfully used to treat HIT [214]. In severe cases and those with an autoimmune component, intravenous immunoglobulin may be considered [215,216,217].

### 5.3. Hypersensitivity Reactions

Hypersensitivity reactions have been reported for ACs [210]. For heparin, the most common hypersensitivity reaction is represented by skin lesions. It has a delayed onset, and it is associated with subcutaneous administration. It can affect 7.5% of users. Skin testing can help confirm the diagnosis [218]. Lesions are often mild, but skin necrosis has also been reported [210]. On the other hand, for coumarin derivatives, hypersensitivity reactions were rare. One of the most severe complications was coumarin necrosis [219]. Warfarin-induced skin necrosis is a rare (0.01–0.1%) but severe complication [220,221]. Researchers are still studying the pathophysiology and involvement of proteins C and S in these processes [222].

Delayed and immediate hypersensitivity reactions were reported for hirudins, including anaphylaxis with a fatal outcome in the case of lepirudin [210], which has been discontinued since 2012 in the USA [219]. Bivalirudin is the only representative on the market due to its low risk of hypersensitivity reactions [223] and can be used in paediatrics [224].

For DOACs, especially rivaroxaban, there were mostly type III and type IV hypersensitivity reactions. Scoring systems (Naranjo score, WHO-UMC Causality categories) are used, but the need for sensitive, cost-efficient and rapid diagnostic tests remains [225].

### 5.4. Anticoagulant Resistance

Heparin resistance is mainly attributed to ATIII deficiency. Heparin sensitivity tests are recommended before surgery [226]. In COVID-19, apparent heparin resistance may reflect hypercoagulable states rather than true ATIII deficiency [227]. Argatroban and hirudin derivatives have been successfully used in cases of heparin resistance [228]. New vitamin K antagonists are being studied to overcome resistance [229]. Warfarin resistance has been reported in humans and rodents due to genetic mutations of vitamin K 2,3-epoxide reductase complex (VKORC) and CYP2C9 [230,231,232]. More than 20 mutations of VKORC were identified in humans [230,233].

### 5.5. Osteopenia and Osteoporosis

Reports on the adverse effects of ACs on bone health are controversial. Some studies concluded that long-term exposure to UFH or warfarin increases the risk of fractures [234,235]. VKAs influence the carboxylation of osteocalcin, resulting in an incompletely gamma-carboxylated form with low affinity. The risk decreases for LMWHs and DOACs [236]. Minimum risk was correlated with the use of direct factor Xa inhibitors. In the latter case, potential benefits regarding bone healing were reported [237]. Novel studies on pharmacogenetics may hold the answer for better risk assessment before starting anticoagulant therapy. VKORC and CYP2D9 polymorphisms have already been documented to have a significant impact on warfarin plasma levels. ABCB1 (ATP-Binding Cassette, Subfamily B, Member 1) and CES1 (Carboxylesterase 1) gene polymorphisms affect plasma levels of DOACs [238].

## 6. Anticoagulants Interactions

### 6.1. Drug–Drug Interactions

Vitamin K antagonists are primarily metabolised by cytochrome P450 enzymes, while DOACs (except dabigatran) are variably affected by CYP3A4, which is a key factor of clinically significant DDIs caused by CYP3A4 inhibitors. These DDIs increase the exposure and bleeding risk, especially in patients taking multiple concomitant drugs [57,76]. In contrast, parenteral anticoagulants (UFH, LMWHs, fondaparinux, bivalirudin, argatroban) have more predictable pharmacokinetics and limited metabolic interactions, with clearance largely dependent on renal or enzymatic pathways rather than the hepatic cytochrome system [77].

For example, a few significant interactions of UFH have been reported. Thus, the studies showed that nitroglycerin decreases the heparin binding of ATIII. To achieve good therapeutic efficacy, UFH doses must be increased. UFH co-administered with aspirin increases bleeding risk [239]. Referring to the LMWHs interactions, a high risk of bleeding in association with non-steroidal anti-inflammatory drugs (NSAIDs), corticosteroids, and piperacillin was reported [240]. Fondaparinux does not present significant interactions with enzymatic inhibitors or inducers, NSAIDs, or other ACs [241].

VKAs’ pharmacokinetics and anticoagulant effects could be altered by changes in bioavailability, as well as adverse effects caused by drug–drug, food–drug, and herbal–drug interactions. Moreover, many medication errors are represented by interactions generated by the concomitant use of VKAs with alternative medicines, especially without informing the prescribers [242,243]. A lot of drug–drug interactions have been reported for VKAs, mainly due to their hepatic metabolization: (i) S-warfarin enantiomer is metabolised by CYP2C9; (ii) R-warfarin enantiomer is metabolised by CYP1A2, CYP2C19, CYP3A4; (iii) acenocoumarol is metabolised by CYP3A4. These enzymatic processes could be inhibited or induced by different drugs or natural compounds. Therefore, some of the main clinical relevant interactions are represented by the inhibition of CYP enzymes, increasing the prothrombin time (PT)/INR and bleeding risk which could be caused by oral azoles such as fluconazole, voriconazole (CYP2C9, CYP3A4, CYP1A2), amiodarone (CYP3A4, CYP1A2), sulfamethoxazole (CYP2C9), metronidazole (CYP2C9), selective serotonin reuptake inhibitors such as citalopram, fluvoxamine (CYP3A4), macrolides such as azithromycin, erythromycin, clarithromycin (CYP3A4), ciprofloxacin (CYP1A2), isoniazid (CYP2C9), piroxicam (CYP3A4), esomeprazole (CYP2C19), etc. [244,245,246,247,248,249]. The induction of hepatic enzymes by anticonvulsants (e.g., phenytoin, carbamazepine, barbiturates), griseofulvin, and rifampicin accelerates the VKA transformation and decreases the anticoagulant effect [250,251,252]. Other pharmacokinetic interactions include displacement of VKAs from plasma proteins by sulfonamides (e.g., sulfamethoxazole) and NSAIDs (e.g., phenylbutazone). Also, cholestyramine reduces the absorption and bioavailability of the VKAs by binding inside the intestinal lumen [253,254,255,256,257]. Some pharmacodynamic interactions produced by VKAs have been reported with (i) AC molecules that have a synergic effect with VKAs; (ii) aspirin that inhibits the platelet function and increases the bleeding risk; (iii) cephalosporins (e.g., 3rd generation) that eliminate the saprophyte bacteria by reducing the intestinal production of vitamin K and increase the bleeding risk by the synergic effect with VKAs; (iv) vitamin K—because of their antagonists’ effects, etc. [258,259].

DOACs’ interactions could be produced by drugs modifying p-glycoprotein from the gut mucosa [248], an ATP-binding cassette transporter (efflux transporter). This protein plays a significant role in drug absorption and distribution [260] and has DOACs as substrate (dabigatran, apixaban, rivaroxaban, betrixaban, edoxaban). Therefore, the association of DOACs with p-glycoprotein inducers (rifampicin, carbamazepine, trazodone, HIV-1 protease inhibitors, amiodarone, digoxin, glucocorticoids, etc.) or inhibitors (e.g., erythromycin, ketoconazole, itraconazole, etc.) is recommended to be avoided or used with caution [244,249]. Rivaroxaban and apixaban are metabolised at a low rate by CYP3A4 and CYP3A5, and the concomitant use with inducers or inhibitors of these enzymes should be avoided [248]. The bleeding risk could be increased because of pharmacodynamic interactions between the DOACs’ association with antiplatelet therapy, NSAIDs, selective serotonin reuptake inhibitors, etc.

### 6.2. Drug–Food Interactions

Some clinical interactions between VKAs and herbs or foods could be considered. Thus, some inhibitors of CYP3A4 (e.g., *Allium sativum*, *Citrus aurantium*, *Citrus paradisi*, *Echinacea* sp., *Eucalyptus globulus*, *Glycyrrhiza glabra*, *Hydrastis canadensis*, *Mentha piperita*, *Prunus avium*, *Silybum marianum*, *Uncaria tomentosa*, *Valeriana officinalis*, etc.), CYP2C9 (e.g., *Allium sativum*, *Citrus bergamia*, *Eucalyptus globulus*, *Mentha piperita*, *Silybum marianum*, *Vaccinium* sp., etc.), CYP2C19 (e.g., *Allium sativum*, *Eucalyptus globulus*, *Mentha piperita*, etc.), or CYP1A2 (e.g., *Mentha piperita*, *Citrus paradisi*, etc.) could increase the anticoagulation activity of VKAs, including the bleeding risk [244,249,261,262,263]. On the other hand, *Hypericum perforatum* is an enzymatic inductor (CYP1A2, CYP2C9, CYP3A4) and increases the VKAs’ clearance, resulting in the reduction of the anticoagulant effect [264].

## 7. Anticoagulant Medication Errors

Although frequently prescribed, UFH remains a difficult drug to manage from medical, nursing, pharmacological, and laboratory perspectives. Medication errors can lead to improper dosing, which could have serious consequences. In some cases, deaths due to UFH overdose have been reported [265,266]. Several studies indicate that patients receiving anticoagulant therapy have not experienced medication errors. For example, Vogel et al. noticed this absence in patients with renal impairment treated with fondaparinux [267].

Table 3 shows several medication errors associated with anticoagulant therapy described in the literature.

**Table 3:** Medication errors of anticoagulant drugs.

Drugs	Type of Error	Consequences	References
VKAs	inadequate prescription	increased risks of bleeding or thrombosis	[128,268]
	inadequate monitoring	increased risk of bleeding	
	poor adherence or inadequate administration	increased risks of bleeding or coagulation due to higher doses, absence of the treatment, or interactions with drugs, plants, food, or dietary supplements	
	continuation of treatment with VKA after switching to other ACs	severe adverse reactions (e.g., bruising with hemorrhagic shock, acute renal failure)	
	continuation of treatment during surgical interventions or invasive manoeuvres	increased risk of bleeding	
	co-administration of acenocoumarol with other drugs (e.g., amiodarone, fluconazole, acetaminophen, acetylsalicylic acid, etc.)	drug–drug interactions which affect the acenocoumarol safety	[269]
DOACs	intermittent drug purchasing due to their high cost by the underinsured or financially unstable patients	increased thrombotic risk	[119]
	inappropriate choice of drug or dose (e.g., in a patient with non-valvular AF, renal impairment, etc.)	thrombotic or bleeding risksoverdoses lead to increased all-cause mortality suboptimal doses lead to increased cardiovascular hospitalizations	[270,271,272]
	concomitant administration with different drug classes (e.g., antiplatelet, nonsteroidal anti-inflammatory drugs)	increased incidence of haemorrhage	[273]
	concomitant administration with CYP3A4 inducers (anticonvulsants, rifampicin, St John’s wort, etc.) or inhibitors (macrolides, antivirals, antifungals, verapamil, amiodarone, etc.)	increased risk of adverse events or reduced efficacy due to modifications in DOAC plasma levels	[274]
	improper storage (e.g., humidity) or administration of DOACs (e.g., crushing of dabigatran tablets, administration of rivaroxaban 20 mg without food, omission of doses, etc.)	reducing the DOACs’ efficiency and increasing the thrombotic risks	[119,275]
	admission or discharge from the hospital, or undergoing surgery	potential or real fatal and serious incidents	[276]
	continuation of treatment during surgical interventions or invasive manoeuvres (e.g., neuraxial anaesthesia) or improper restarting of therapy after these interventions	bleeding complications (e.g., spinal or epidural hematoma) or thrombotic risks	[277]
	duplicate prescribing, incorrect dispensing, incorrect prescription (frequency, wrong patient, dose, duration)	N/A	[278]
	wrong drug or dose (e.g., in patients with impaired renal function), duplicate therapy, missing drug/omission because of the patients’ discharge without anticoagulation, failure to restart DOACs post-procedure, or low adherence	N/A	[279,280]
	inappropriately low or high dose	tendency of adverse events (stroke, transitory ischemic accident, embolism, bleeding complications), but not statistically significant	[281]
	incorrect dosing (e.g., in patients with impaired renal function, elderly or treated with other drugs such as verapamil), intentional dose reducing because of doctors’ fear of bleeding risk, off-label prescriptions (e.g., bariatric surgery)	reducing drug safety reducing the drug efficacy and increasing the risk of thrombotic events	[282]
	the overdosing was reported in the EudraVigilance database with a higher frequency than underdosing	the correct usage of DOACs should be monitored by professionals	[283]
	under-dosing in elderly patients or in patients with a higher CHA_2_DS_2_-VASc score, over-dosing in elderly patients or in patients with renal failure or a higher bleeding score	higher all-cause mortality (overdoses) frequent hospitalisation for cardiovascular problems, such as stroke (underdoses)	[284,285]
UFH	accidental administration of heparin saline solution rather than heparin	low or lack of efficacy	[286]
	coexistence of many protocols in the same medical site for the administration of UFH in continuous infusion	N/A	[287]
	administrative errors (inaccurate filling of automatic drug-dispensing cabinets), similar sizes of vials or labels, incorrect dosing of factor X, and inaccuracy in label reading	severe conditions in neonates (including death)	[288]
UFH after DOACs therapy	falsely elevated anti-Xa concentrations and incorrect titration based on anti-Xa concentrations in hospitalised patients converted from DOAC to UFH	thrombotic events, death	[289]
UFH and LMWHs	prescribing protocol problems	increased risk of incidence	[290]
	dosing and monitoring confusion, use of abbreviations, dosage calculations, multiple-solution concentrations, use of intravenous delivery pumps	bleeding complications	[265]
LMWHs	incomplete prescriptions, omission errors (the ordered drug is not given), performance deficit of the medical team, and failure to follow protocols and guidelines	reducing the drug’s safety or efficacy	[291]
	ignoring the patient’s condition. LMWH were inadequately administered in patients with renal failure, history of HIT, and stents after surgery (incorrect post-surgery co-administration of enoxaparin, aspirin, and clopidogrel)	bleeding complications, death platelet count decreased, and the PF4 antibody was positive, cyanosis, reduced oxygen saturation, and death total stenosis of the coronary arteries	[266]
	misuse of devices and incorrect programming of infusion devices	overdose and bleeding complications (e.g., bleeding at the administration site)	[266]
	mismanagement (enoxaparin) of the route of administration in neonates (i.m. administration, not s.c.)	increased aPTT, high bleeding risk	[292]
	dosing errors (dose ordered was not based on weight) or duplicate therapy errors (co-administration of UFH and LMWH)	N/A	[293]
Parenteral direct thrombin inhibitors	prescribing and administration errors, delayed dose adjustment after aPTT determination delayed infusion rate adjustment when clinical pharmacy staff are less available (e.g., evenings, weekends, etc.)	N/A	[294]
	dispensing of a wrong drug (e.g., tirofiban instead of argatroban)	Lack of efficacy	[294]
	administration of argatroban in the wrong patients	N/A	[295]

N/A—not available; ACs—anticoagulant drugs; AF—atrial fibrillation; aPTT—activated partial thromboplastin time; CHA_2_DS_2_-VASc—congestive heart failure, hypertension, age, diabetes mellitus, prior stroke or transient ischemic attack or thromboembolism, vascular disease, age, sex category; DOACs—direct oral anticoagulant drugs; HIT—heparin-induced thrombocytopenia; i.m.—intramuscular; LMWHs—low-molecular-weight heparins; s.c.—subcutaneous; UFH—unfractionated heparin; VKAs—vitamin K antagonists.

In order to reduce medication errors, specialists have elaborated some protocols or guidelines that contain the main elements responsible for these errors, such as patient condition, drug use (including protocols, doses, etc.), adequate communication of the main issues to the patient (including phone application, handwriting, avoiding the ambiguous abbreviations, etc.), staff and patient education, drug manufacturing (including labelling, packaging, storage, etc.), using adequate drug devices, etc. [266,296].

A high number of cancelled surgical procedures have been reported due to inadequate patient preparation and incorrect management of anticoagulant therapy (e.g., acenocoumarol, enoxaparin, DOACs, etc.) [297]. Many studies have shown the improvement of therapeutic or surgical conditions due to the involvement of pharmacists in the management of medication errors, and in avoiding the cancellation of surgical procedures because of incorrect drug management [279,294,297].

## 8. Reversal Anticoagulant Agents

The management of major bleeding in patients treated with ACs is guided by the aetiology and clinical manifestations. Major bleeding can affect one or more areas or organs, lead to a decrease in haemoglobin level by ≥20 g/L, and can be fatal. AC treatment must be discontinued, and the bleeding site and hemodynamic stability must be established. Subsequently, some general measures should be taken: (i) maintaining adequate fluid, oxygen, and hemodynamic support; (ii) platelet transfusion; (iii) using cryoprecipitate in patients with hypofibrinogenemia; (iv) activated charcoal in patients treated with DOACs; (v) dialysis in patients with renal disease treated with dabigatran; (vi) using reversal agents (Table 4) [207,298].

During these interventions, monitoring of the residual effect of ACs should be performed [207,298]:

- aPTT: UFH, LMWHs, dabigatran;
- Prothrombin time/INR (PT/INR): VKAs;
- Ecarin clotting time (ECT): dabigatran;
- Hemoclot thrombin inhibitor assay: dabigatran;
- Thrombin time: dabigatran;
- Anti-factor Xa level: LMWHs, fondaparinux, direct factor Xa inhibitors.

**Table 4:** Reversal agents for anticoagulant drugs [207,298,299].

Reversal Agent	Anticoagulant Drug	Posology
Protamine sulfate	UFH, LMWHs	12.5–50 mg i.v.
Vitamin K	VKAs	1–10 mg i.v. or p.o.
Idarucizumab	dabigatran	2 × 2.5 g i.v.
Andexanet alfa	direct factor Xa inhibitors LMWHs *, fondaparinux *	0.4 or 0.8 g i.v. bolus followed by a continuous infusion of 4 mg/min or 8 mg/min for up to 120 min
Tranexamic acid	VKAs direct factor Xa inhibitors	1 g at 8 h
FFP	VKAs DOACs ***	10–15 mL/kg
PCC ^#^	VKAs	25–50 units/kg
rFVIIa	VKAs dabigatran ***	90 µg/kg
Activated charcoal	VKAs DOACs argatroban and lepirudin	50–100 mg/dose
Hemodialysis ^##^	dabigatran	
Aripazine (ciraparantag)	UH, LMWHs, fondaparinux, DOACs	25 mg, 50 mg, 100 mg, 300 mg, and 600 mg i.v. **

DOACs—direct oral anticoagulant drugs; i.v.—intravenous; LMWHs—low-molecular-weight heparins; p.o.—oral; rFVIIa—recombinant-activated factor VIIa; UFH—unfractionated heparin; VKAs—vitamin K antagonists. ^#^ PCC (prothrombin complex concentrate) should contain four coagulation factors (II, VII, IX, X) or a minimum of 3 (II, IX, X). ^##^ In patients with renal impairment. * Treatment with andexanet alfa is not approved for bleeding caused by LMWHs or fondaparinux. ** The efficiency of aripazine at different doses is under investigation. *** FFP (fresh frozen plasma) should be considered when other reversal agents are unavailable.

### 8.1. Protamine Sulfate

Protamine sulfate is produced by recombinant technology from salmon sperm and could be administered as a slow intravenous bolus to prevent the interaction between heparin and ATIII and reverse the anticoagulant effect. In combination with heparin, it generates an inactive aggregate (heparin–protamine salt aggregate) and cancels the heparin anticoagulant effect [298]. One mg of protamine could neutralise 80–100 units of heparin. Because protamine is less efficient at reversing bleeding induced by LMWHs (enoxaparin, dalteparin, and tinzaparin), it is not approved by the FDA. Protamine is not efficient in reversing the effects of fondaparinux or idraparinux. A major disadvantage of protamine is its potential for anaphylactic reactions, including hypotension, bradycardia, bronchoconstriction, etc. [102,207,300,301].

### 8.2. Vitamin K

Vitamin K represents the specific reversal agent used in bleeding caused by VKAs (INR > 10) or in patients with bleeding risk factors (INR value between 4.5 and 10). The effect of VKAs is reversed within 24–48 h after oral administration or within 12–14 h after i.v. administration of 1–10 mg vitamin K [207]. VKAs’ toxicity can result from intentional or unintentional adult overdose, paediatric ingestion, or association of VKAs with other drugs which increase toxicity (including association with illicit drugs to prolong euphoric effects) [302].

### 8.3. Idarucizumab

Idarucizumab is a small fragment of a humanised monoclonal antibody that binds dabigatran (including its metabolites) and rapidly and completely reverses its effects (half-life of idarucizumab is about 45 min). Within a maximum of 30 min, it normalised laboratory measures (dilute thrombin time (dTT) or ECT in 90% of patients treated with two consecutive doses of idarucizumab (2.5 g/dose) administered i.v. [303,304,305].

### 8.4. Andexanet Alfa

Andexanet alfa is a sequestrant of direct factor Xa inhibitors (apixaban, rivaroxaban, edoxaban) and is used to reverse their effects. It is a recombinant-modified human factor Xa that decreases anti-factor Xa activity (up to 88%) after bolus administration, followed by i.v. infusion (0.4–0.8 g/min) for a maximum of 2 h. No thrombotic complications have been reported with andexanet. At the moment, it is not approved for reversing the LMWHs or fondaparinux activity [14,207,299,306].

### 8.5. Fresh Frozen Plasma

Fresh frozen plasma (FFP) is considered the elective treatment for decreasing INR after VKAs treatment (10–15 mL/kg) and contains all coagulation factors from the blood [307]. FFP should be considered for the reversal of DOACs in an extreme situation when other reversal agents are unavailable. Some disadvantages limit FFP administration: (i) ABO compatibility; (ii) the need for thawing before administration; (iii) a long time for INR reversing (6–24 h); (iv) the risk of volume overload; (v) the risk of transfusion-related acute lung injury [26,307].

Cryoprecipitate is a concentrate obtained by centrifugation of slowly thawed FFP. The precipitate resulting from this procedure contains high-molecular-weight plasma proteins: fibrinogen, factor VIII, von Willebrand factor, factor XIII, and fibronectin. Its main indications are hypofibrinogenemia, haemophilia, von Willebrand disease, bleeding from excessive anticoagulation, etc. [308]. The use of cryoprecipitate is limited by its adverse events (transmission of blood-borne pathogens, transfusion-related acute lung injury). Moreover, in many countries, it has been withdrawn from uses [309].

### 8.6. Prothrombin Complex Concentrate

Prothrombin Complex Concentrate (PCC) is mainly used off-label (25–50 units/kg) in patients treated with VKAs for quick INR reversal (15–20 min). It is obtained from human plasma and generally contains four coagulation factors (II, VII, IX, X). To prevent the activation of endogenous coagulation pathways, anticoagulant molecules (e.g., protein C, protein S, heparin, antithrombin III, etc.) could be found in the PCC composition [26,307]. Despite their high cost, PCC is preferred to FFP due to the absence of volume overload and the risks of acute lung injury [207]. In addition to specific treatment with vitamin K, patients with bleeding after VKAs treatment could receive PCC with four factors (II, VII, IX, X), PCC with three factors (II, IX, X) and FFP, or only FFP [300,302]. The four-factor PCC could be used as a specific reversal strategy in patients treated with direct factor Xa inhibitors [300].

### 8.7. Recombinant-Activated Factor VIIa

Recombinant-activated factor VIIa (rFVIIa) activates the coagulation cascade via the extrinsic pathway and could be used off-label for INR correction in VKA overdose. Although it normalises the INR quickly during the induction of a thrombin burst, in patients treated with VKAs, rFVIIa is an unreliable measure of the antithrombotic effect. PT/INR is very sensitive to the depletion of factor VII. At steady state, there is an equilibrium between the depletion of factors II and X, and on the other hand, the depletion of factor VII. PT/INR monitoring is non-specific and exhibits significant biological variability [307,310]. Also, rFVIIa may be beneficial in uncontrolled bleeding induced by fondaparinux or idraparinux [102].

In an experimental model, rFVIIa normalised haemostasis time after therapeutic doses of dabigatran by reducing thrombin lag time and increasing thrombin generation [311]. Compared to activated PCC, a low efficiency of rFVIIa in reversing the anticoagulant effect of rivaroxaban was obtained [312].

### 8.8. Activated Charcoal

In patients who are awake and alert, activated charcoal can be administered within the first hour after VKA ingestion (100 mg orally) [300,302]. In vitro studies showed the efficacy of activated charcoal in removing DOACs from the body (after 8 h for rivaroxaban, 6 h for apixaban, and up to 2 h for edoxaban and dabigatran) and its ability to bind to argatroban and lepirudin when administered intravenously. On the other hand, no effect after heparin administration was observed [26,311,313,314].

### 8.9. Haemodialysis

Haemodialysis is efficient at removing dabigatran, which has a low plasma protein binding. Because direct factor Xa inhibitors are highly bound to plasma proteins, these drugs cannot be removed by haemodialysis [311].

### 8.10. Other Strategies

#### 8.10.1. Tranexamic Acid

Tranexamic acid is mainly used to treat bleeding caused by various traumas. It improves platelet function by inhibiting fibrinolysis (the conversion of plasminogen into plasmin). Also, i.v. administration (1 g every 8 h) in patients with bleeding after VKAs or direct factor Xa inhibitors treatment should be considered [300].

#### 8.10.2. Aripazine (Ciraparantag)

Aripazine is a synthetic molecule (di-arginine piperazine derivative) under investigation, developed as a “universal” reversal agent of the anticoagulant effect induced by UFH, LMWHs, fondaparinux, or DOACs. Studies showed that it binds to DOACs (hydrogen bonds). For example, in a clinical trial, aripazine administered in a single dose reversed the blood clotting time modified by edoxaban, within 10 min [207,315].

## 9. Originals Versus Biosimilars

LMWHs are considered biological drugs according to European regulations and semisynthetic drugs according to US regulations. Therefore, EMA included their copies in the biosimilars category and FDA in the generic drugs category. According to this classification, for the market approval of biosimilars, it is necessary to present the results of clinical trials, not only of in vivo pharmacodynamic studies [316].

Loss of the patent for the original LMWHs was a significant concern regarding biosimilars’ efficacy and safety. Enoxaparin became the gold standard in cardiology due to its higher efficacy compared with UFH or other LMWHs. According to many clinicians, an equivalent benefit/risk ratio of biosimilar versus original enoxaparin might be challenging to demonstrate [317].

For the characterisation of these molecules (especially enoxaparin and enoxaparin biosimilars), many methods were proposed: (i) mass spectrometry used to detect the labile sulfates attached to the side-groups of LMWH [318], (ii) strong anion-exchange chromatography with spectrophotometric detection [319], (iii) LC-MS and NMR for the characterisation of homogeneous octasaccharide and decasaccharide components together with their fractions endowed with high or no affinity for ATIII [320], etc.

Another study demonstrated the pharmacokinetic and pharmacodynamic equivalence of a biosimilar enoxaparin to the original in healthy volunteers [321]. However, despite clinicians’ concerns, some studies suggest that biosimilar enoxaparin is a potential alternative to the original products, due to its efficacy (e.g., anti-Xa/anti IIa: 3.9, activated clotting time, aPTT, and thrombin time (TT)) and low incidence of adverse reactions (bleeding) [322,323,324].

An octadecasaccharide containing three ATIII sites and a very high antithrombotic activity (anti-factor Xa) was isolated and purified. It could offer a new perspective on the modulation of thrombin activity [325]. Moreover, to confirm the efficacy and safety of LMWH biosimilars, proactive pharmacovigilance monitoring is recommended [326].

## 10. Laboratory Monitoring of Anticoagulant Drugs

To reduce the risk of bleeding with ACs, monitoring anticoagulant therapy is mandatory in many cases. Most laboratory tests used include the PT/INR ratio and aPTT, chromogenic anti-Xa assay, and ECT (Table 5).

Prothrombin time or Quick’s Prothrombin Time (PT) represents the time needed for plasma to clot in the presence of calcium and tissular thromboplastin equivalent reagent. Because it assesses extrinsic and common coagulation pathways, it is influenced by the activity of some clotting factors (II, V, VII, X) and fibrinogen. It is increased by VKAs, UFH (high doses), DOACs, and parenteral direct inhibitors of clotting factor IIa [33,340].

The INR is calculated based on the following formula [33,328]:

INR = (PT test/MNPT)^ISI^

MNPT (mean normal PT) or PT control is obtained from a minimum of 20 healthy persons, and ISI represents the international sensitivity index (the ability of thromboplastin to reduce vitamin-K-dependent factor compared to a standard thromboplastin).

aPTT represents the time needed for recalcified plasma to clot through adding a platelet phospholipid generator and a surface-activating agent (e.g., celite, ellagic acid, kaolin, or micronised silica). It determines the activity of intrinsic and common coagulation pathways and is used mainly to monitor UFH and parenteral direct inhibitors of clotting factor IIa, which prolong the aPTT. Also, it is used as a general test for the state of anticoagulation [33,328].

Thrombin time (TT) represents the clotting time of the plasma that contains AC. Clotting is produced by excess thrombin added to the plasma. TT can be prolonged by dabigatran, and it is the most sensitive assay for detecting dabigatran presence. VKAs produce a non-significant increase [341,342]. The diluted TT assay (dTT) is used for dabigatran monitoring and as an additional test for monitoring parenteral direct inhibitors of thrombin [336,343].

An alternative to the aPTT test for monitoring UFH and LMWHs is the prothrombinase-induced clotting test (PiCT). This test contains factor X, phospholipids, and an enzyme from viper venom as a specific activator of factor V. After incubation in the presence of CaCl_2_, a prothrombinase complex is formed. This complex initiates the coagulation cascade by activating factor X. The test is sensitive to inhibitors of factors IIa and Xa, too [329,344,345].

The Dilute Rusell Viper Venom Time assay (dRVVT) contains an activator of factor X. In the presence of phospholipids, prothrombin, and Ca^2+^, it produces clotting through the conversion of prothrombin into thrombin and then of fibrinogen into fibrin. The test is used to detect lupus anticoagulants because the complex formed between phospholipids and antibodies inhibits venom activity. As a result, the clotting time is prolonged [346].

Anti-FXa activity measures, through a colourimetric method, the ATIII-catalysed inhibition of factor Xa activity from the reagent by different ACs (UFH, LMWH, fondaparinux, or heparin analogues) [347]. One of the various anti-FXa activity assays is HepTest, which contains a mixture of CaCl_2_ and brain cephalin in a bovine plasma fraction [348].

Activated clotting time (ACT) determines the inhibitory effect of heparin during medical or surgical procedures. A prolonged clotting time shows a higher degree of anticoagulation. This test is used for monitoring of parenteral thrombin inhibitors [349].

ECT is a chromogenic anti-IIa assay. Prothrombin is converted to meizothrombin by ecarin, a metalloprotease obtained from snake venom. Direct inhibitors of thrombin, such as hirudin and its analogues, neutralise meizothrombin and prolong ECT [328,350].

Rotational thromboelastometry (ROTEM) is a method used to quantify coagulation parameters through different tests: (i) measurement of aPTT and evaluation of the intrinsic pathway (INTEM); (ii) measurement of Quick time (PT) and evaluation of the extrinsic pathway (EXTEM); (iii) measurement of TT and evaluation of fibrinogen status (FIBTEM) [350].

Thromboelastography is a non-invasive test. The changes in the viscoelasticity of integral blood are quantified [351]. It could be used to monitor DOAC treatment by measuring clot parameters such as reaction time (r-time), coagulation time (k-time), maximum amplitude, and angle α formed by the slope of a tangent line traced from r-time to k-time. TEG with ecarin is used to monitor hirudin and its analogues [338,339].

D-dimers are fibrin degradation products used as markers in the diagnosis of various pathologies, including VTE. In anticoagulant therapy, this laboratory test could be used to evaluate the progress of anticoagulation and to determine the optimal duration or intensity of anticoagulation, especially in patients with mechanical heart valve replacement [337,352,353].

To initiate treatment with UFH, LMWH, and ULMWH, some tests are recommended: platelet count, aPTT, and INR. For monitoring the therapy with LMWH, ULMWH, and fondaparinux, other tests are described in the literature: (i) thrombin generation time, which represents the speed of thrombin generation; (ii) platelet contractile force, which determines the platelet function during clotting, and (iii) clot elastic modulus, which shows the clot integrity [354].

## 11. Future Perspectives

One of the current directions in anticoagulant therapy is exploring alternative routes of administration for existing anticoagulants. Thus, UFH is being investigated for topical administration, while LMWHs are studied for oral, pulmonary, transdermal, or topical delivery [47].

Moreover, several studies have evaluated bioactive compounds with anticoagulant properties. An in vivo study using a nanogel based on Pluronic P123 and heparin, loaded with adenosine and the dipeptide Ile–Trp, two compounds extracted from scorpion venom (*Heterometrus laoticus*), showed a significant prolongation of clotting and bleeding times compared with free bioactive compounds [355]. In parallel, green chemistry has gained increasing importance in pharmacotherapeutics, aiming to reduce the negative impact of hazardous chemicals [356]. Mono- and bimetallic nanoparticles represent a significant focus of anticoagulant research [357], with particular interest in biogenic metallic nanoparticles due to their improved biocompatibility, efficiency, and reduced required doses [358]. For example, silver nanoparticles bound to *Selaginella bryopteris* were shown to affect the intrinsic coagulation pathway, as evidenced by prolonged aPTT without changes in PT [357]. Similarly, silver nanoparticles biosynthesised using *Petiveria alliacea* leaf extract prevented blood coagulation [358]. Comparable effects were observed with heparin-reduced gold nanoparticles, which prolonged PT, TT, and aPTT compared with heparin alone [359].

Alongside advances in understanding the biochemical mechanisms of coagulation, more selective inhibitors targeting specific coagulation factors have been developed, showing improved efficacy and safety in VT [243]. Several studies have demonstrated that factor XI plays a limited role in physiological (secondary) haemostasis but is crucial in VT. Consequently, factor XI has emerged as a promising target for anticoagulant therapy, with inhibition considered safer than targeting factors II or X [360,361]. This category includes inhibitors of factor XI, either by blocking its biosynthesis or preventing its activation, such as small molecules, antisense oligonucleotides, and monoclonal antibodies [362,363]. Examples of factor XI inhibitors currently under investigation are presented in Table 6.

Another category of molecules studied as anticoagulants is represented by aptamers. Aptamers are a class of single-stranded oligonucleotides, typically 25–80 bases in length, isolated from combinatorial libraries of nucleic acids using an in vitro selection process known as systematic evolution of ligands by exponential enrichment (SELEX). These compounds can bind specific target molecules with affinities comparable to those of monoclonal antibodies (mAbs) [378].

Compared with mAbs, aptamers offer several advantages. The traditional development of mAbs involves successive immunisations of a host animal, followed by the isolation of antibody-producing cells and their fusion with rapidly replicating myeloma cells. Subsequent stages include hybridoma selection and antibody production under physiological conditions. Each step is time-consuming, and the entire process typically takes one to three months. Moreover, aptamers can be selected to function under specific buffer conditions or in the presence of particular proteins or chemical compounds. Aptamers have demonstrated improved tissue penetration under both in vivo and in vitro conditions [379].

Thrombin aptamers are considerably smaller molecules than mAbs (approximately 25 Å/21 Å compared with approximately 139 Å/122 Å) [380] and present multiple advantages, including prolonged functional lifetime, very low immunogenicity, and the feasibility of chemical modifications to enhance stability, extend half-life, and enable targeted delivery. The most effective aptamers are those in bivalent or circular forms, which have demonstrated improved in vivo stability and more efficient accumulation and retention compared with monoaptamers [378]. NU172 (ARC2172) is an unmodified deoxyribonucleic acid aptamer that binds to thrombin exosite I. It has a short in vivo half-life of approximately 10 min in circulation [381]. This aptamer demonstrated dose-dependent anticoagulant activity without significant adverse effects [382,383].

In addition, various non-anticoagulant heparin derivatives have been obtained through chemical modifications, such as depolymerisation, oversulfation, N-desulfation, O-desulfation, carboxyl substitution or reduction, and glycol splitting of uronic acid moieties [384]. For example, Roy et al. obtained a new series of partially desulfated heparin derivatives and investigated their biological activity using in vitro and in vivo models. These derivatives exhibited reduced anti-factor Xa activity but showed varying affinities for angiogenic growth factors, including vascular endothelial growth factor (VEGF), stromal cell-derived factor-1α (SDF-1α), and fibroblast growth factor-2, depending on the site and degree of desulfation. The study indicated that a trisulfated disaccharide content above 35%, regardless of O- or N-position, is necessary to maintain biological activity, as higher degrees of desulfation were associated with a significant reduction in activity [385]. Furthermore, Pan et al. developed a synthetic heparosan-type heptasaccharide with potent anti-inflammatory activity following partial desulfation and subsequent fractionation of low-molecular-weight heparin. A negative correlation between the degree of sulfation and the anti-inflammatory effect was observed [386].

Thus, the new generation anticoagulants are designed with a much more selective mechanism of action in order to reduce bleeding risk and increase their antithrombotic efficacy. Future research will focus on upstream coagulation factors such as factor XI (mAbs, small synthetic molecules, antisense oligonucleotides), as well as new molecular platforms (e.g., aptamers, nanoparticles) and modified heparin derivatives with specific biological properties. The results from preclinical and early clinical data are promising, but some challenges, such as long-term safety, reversibility of effects, large-scale manufacturing, and high production costs, still need to be addressed. Once these shortcomings are overcome, these innovative approaches will be viable for extensive clinical use.

## 12. Conclusions

Anticoagulant therapy is vital for preventing thrombosis but carries significant risks, including bleeding, heparin-induced thrombocytopenia, hypersensitivity, and drug interactions. Effective management requires balancing these risks through individualised dosing, careful monitoring, and the use of reversal strategies and laboratory tests. Despite the high effectiveness of classic anticoagulants and the presence of adverse reactions, the need to improve treatment adherence and reduce medication errors has led to the development of new classes of anticoagulants. While DOACs and biosimilar LMWHs offer high efficacy, research is shifting toward alternative delivery methods, selective factor XI inhibitors, aptamers, and modified heparins to enhance efficacy while minimising bleeding risks. Overall, anticoagulant therapy relies on balancing thrombotic prevention with haemorrhagic risk through evidence-based strategies and ongoing innovation.

## Figures and Tables

**Table 1:** Classification of anticoagulant drugs [10,27,32,33,34,35,36,37,38,39,40].

Class	Mechanism of Action	Drugs	Observations
1. Indirect inhibitors of clotting factors IIa and Xa			
Unfractioned heparin (UFH)	indirect inhibition of the clotting factor IIa (thrombin) and Xa, by interaction with ATIII (enhances the affinity of ATIII for both clotting factors Xa and IIa)	Heparin	Mean MW: 15,000 kDa (range, 3000–30,000)
Low-molecular-weight heparins (LMWHs)	indirect inhibition of the clotting factors Xa and less IIa (thrombin), by interaction with ATIII (enhances the affinity of ATIII for both clotting factors Xa and IIa)	Ardeparin **	MW: 5.5–6.5 kDa, anti-Xa/anti IIa: 1.7–2.4
		Dalteparin	MW: 6.0 kDa, anti-Xa/anti IIa: 2.5
		Enoxaparin	MW: 4.5 kDa, anti-Xa/anti IIa: 3.9
		Nadroparin ***	MW: 4.3 kDa, anti-Xa/anti IIa: 3.3
		Tinzaparin	MW: 6.5 kDa, anti-Xa/anti IIa: 2.6
		Reviparin	MW: 4.4 kDa, anti-Xa/anti IIa: 4.2
		Parnaparin	MW: 5.0 kDa, anti-Xa/anti IIa: 2.3
		Certoparin	MW: 5.4 kDa, anti-Xa/anti IIa: 2.4
Ultra-low-molecular-weight heparins (ULMWHs)	indirect inhibition of the clotting factors Xa and less IIa (thrombin), by interaction with ATIII (enhances the affinity of ATIII for both clotting factors Xa and IIa)	Bemiparin	MW: 3.6 kDa, anti-Xa/anti IIa: 9.7
		Semuloparin **	MW: 2.4 kDa, anti-Xa/anti IIa: 80
		Deligoparin **	
Indirect inhibitors of the activity of clotting factor Xa	indirect inhibition of clotting factor Xa (enhances the affinity of ATIII for clotting factor Xa)	Fondaparinux	MW: 1728 gmol, anti-Xa/anti IIa: ∞
		Idraparinux **	
		Idrabiotaparinux **	
Heparinoids	indirect inhibition of the clotting factor Xa (enhances the affinity of ATIII for clotting factor Xa)	Danaparoid	contains heparan sulfate (84%), dermatan sulfate (12%), chondroitin sulfate (4%); MW: 6.0 kDa anti-Xa/anti IIa > 20
		Pentosan polysulfate	semisynthetic polysaccharide derived from beech tree bark MW: 6.0 kDa
2. Direct inhibitors of clotting factors IIa and Xa			
Parenteral direct thrombin inhibitors	direct inhibition of clotting factor IIa (specific thrombin inhibitor)	Argatroban	MW: 527Da
		Desirudin	
		Lepirudin **	Recombinant hirudin
		Bivalirudin	Synthetic congener of hirudin
Direct oral inhibitors of clotting factor IIa (DOAC)	direct inhibition of clotting factor IIa (specific thrombin inhibitor)	Dabigatran etexilate	Prodrug
		Ximelagatran **	Prodrug
		Melagatran **	Active form of ximelagatran
Direct oral inhibitors of clotting factor Xa (DOAC)	direct inhibition of clotting factor Xa	Rivaroxaban	
		Apixaban	
		Edoxaban	
		Betrixaban	
3. Inhibitors of the hepatic synthesis of clotting factors			
Vitamin K antagonists (VKAs)	Inhibition of hepatic synthesis of some clotting factors by antagonising vitamin K as a cofactor for synthesis cofactor	Warfarin	Racemic (R, S *)
		Acenocumarol (nicumalone)	Racemic (R *, S)
		Dicumarol	
		Phenprocoumon **	Racemic (R, S *)
		Tioclomarol **	
		Ethyl biscoumacetate **	
		Phenindione	Higher incidence of severe adverse effects
		Anisindione	
		Fluindione	Non-chiral

MW: molecular weight; * the most active enantiomer; ** experimental, or not approved for therapeutic use, or withdrawn from the market; *** all LMWHs are available as sodium salts, except nadroparin, which is available as a calcium salt.

**Table 5:** Laboratory tests used in anticoagulant therapy monitoring.

Drug Class	Monitoring Laboratory Test	Observation	References
1. Indirect inhibitors of clotting factors IIa and Xa			
UFH	aPTT *, TT, PiCT, ACT (high doses of UFH), Anti-Xa assay, HepTest	aPTT: 1.5–3.5 b.v. Anti-Xa: 0.2–0.6 I.U./mL PiCT: 77–110 s decrease in D-dimer levels Platelet counts are decreased in patients with HIT	[33,327,328,329,330]
LMWHs and ULMWHs	Anti-Xa assay *, HepTest, PiCT, aPTT		[40,328]
Fondaparinux	no monitoring required (PiCT **, Anti-Xa assay **, HepTest **, aPTT **)		[40,85,328,331]
Heparinoids	Anti-Xa assay		[332,333]
2. Direct inhibitors of clotting factors IIa and Xa			
Parenteral direct thrombin inhibitors	ACT *, aPTT *, TT, DTT, ECT, ROTEM (in critically ill patients), chromogenic anti-Iia	aPTT: ×1.5 to 2 b.v. (lepirudin) ×1.5 to 3.0 b.v. (argatroban); ×1.5 to 2.5 b.v. (bivalirudin)	[85,328,334,335,336]
Direct oral anticoagulant inhibitors of clotting factor IIa	DTT *, ECT *, Anti-II a assay *, TT (oversensitive), PT/INR (low sensitive), aPTT ***, dRVVT ****	decrease in D-dimer levels	[328,337]
Direct oral anticoagulant inhibitors of clotting factor Xa	chromogenic anti-Xa assays *, HepTest ACT, thromboelastography **, ROTEM **, PT ***, aPTT ***, PiCT, dRVVT ****	decrease in D-dimer levels	[328,337,338,339]
3. Inhibitors of the hepatic synthesis of clotting factors			
VKAs	PT/INR *, aPTT	INR: 2.0–3.0 decrease in D-dimer levels	[33,328,337]

aPTT—activated partial thromboplastin time; ACT—activated clotting time; b.v.—baseline values; dRVVT—dilute Russell viper venom time; DTT—dilute thrombin time; ECT—ecarin clotting time; INR—international normalized ratio; ROTEM—rotational thromboelastometry; TT—thrombin time; PT—prothrombin time; PiCT—prothrombinase-induced clotting time; *—the main standard for monitoring; **—optional; ***—unpredictable; ****—under investigation.

**Table 6:** Inhibitors of factor XI. aPTT—activated partial thromboplastin time; mAb—monoclonal antibodies; mRNA—messenger RNA; s.c.—subcutaneous, i.v.—intravenous.

Compound	Administration Route and Frequency	Observations	Reference
Monoclonal antibodies (mAb)			
Abelacimab (MAA868)	s.c./i.v. once per month	binds the catalytic domain of FXI and prevents its activation (tested in Phase III)	[364,365,366,367]
BAY 1831865	s.c./i.v. once per month	very good tolerance, no signs of bleeding, pronounced, sustained, dose-dependent prolongation of the duration of factor XI inhibition.	[368]
Osocimab (BAY 1213790)	s.c./i.v.	favourable safety and tolerability; no bleeding or antibody formation was observed	[367,369,370]
AB023 (Xisomab 3G3)	i.v.	a single administration of 1.0 mg/kg compound reduced total platelet aggregation and total fibrin deposition in vascular grafts	[367,371]
Small synthetic molecules			
Asundexian (BAY 2433334)	oral	fast onset of action, well tolerated, with a predictable pharmacokinetic/pharmacodynamic profile and no clinically relevant induction or inhibition of CYP3A4	[367,372,373]
ONO-7684	oral	well tolerated at all tested dose levels, with an overall low incidence of adverse treatment reactions	[374]
SHR2285	Oral	safety, pharmacokinetic, and pharmacodynamic profiles in the 50 mg–400 mg dose range.	[375]
Milvexian (JNJ-70033093/BMS-986177)	Oral	direct active-site inhibitor of FXIa; milvexian has been evaluated for the prevention and in vivo treatment of VT.	[367,376]
Antisense oligonucleotides			
Fesomersen	s.c.	a dose-dependent reduction in hepatic FXI mRNA expression and prolongation of aPTT	[367,377]
(IONIS-FRIRX-LRx/	s.c.	reduction of hepatic synthesis of FXI	[363]

## Data Availability

No new data were created or analyzed in this study. Data sharing is not applicable to this article.

## Abbreviations

ABCB1	ATP-binding cassette, subfamily b, member 1
AC	anticoagulant drug
ACT	activated clotting time
AF	atrial fibrillation
APS	antiphospholipid syndrome
aPTT	activated partial thromboplastin time
ATIII	antithrombin III
b.v.	baseline values
BID	bis in die (twice a day)
CES1	carboxylesterase 1
CHA_2_DS_2_-VASc	congestive heart failure, hypertension, age, diabetes mellitus, prior stroke or transient ischemic attack or thromboembolism, vascular disease, age, sex category (score)
CKD	chronic kidney disease
CYP	cytochrome P450
DOAC	direct oral anticoagulant drug
dRVVT	dilute Rusell viper venom time
dTT	dilute thrombin time
DVT	deep venous thrombosis
ECT	ecarin clotting time
FDA	Food & Drug Administration
FFP	fresh frozen plasma
HIT	heparin induced thrombocytopenia
i.m.	intramuscular
i.v.	intravenous
INR	international normalized ratio
IU	Internation Unit
k-time	coagulation time
LMWH	low-molecular-weight heparin
mAb	monoclonal antibody
MAPK	mitogen-activated protein kinase
MNPT	mean normal PT
mRNA	messenger RNA
MW	molecular weight
N/A	not available
NOAC	novel or non-vitamin K antagonist oral anticoagulant
NSAIDs	non-steroidal anti-inflammatory drugs
NSTEMI	non-ST-elevation myocardial infarction
p.o.	per os (oral)
PCC	prothrombin complex concentrate
PCI	percutaneous coronary intervention
PE	pulmonary embolism
PiCT	prothrombinase-induced clotting test
PIVKA	proteins induced by vitamin K absence
PT	prothrombin time
QD	quaque die (once a day)
R	renal elimination
rFVIIa	recombinant-activated factor VIIa
ROTEM	rotational thromboelastometry
r-time	reaction time
s.c.	subcutaneous
SDF-1	stromal cell-derived factor-1α
STEMI	ST-elevation myocardial infarction
TID	ter in die (three times a day)
TSOAC	target-specific oral anticoagulants
TT	thrombin time
TTR	time in therapeutic range
UFH	unfractioned heparin
ULMWH	first ultra-low-molecular-weight heparin
USD	United States dollar
VEGF	vascular endothelial growth factor
VKA	vitamin K antagonist
VKOR	vitamin K oxide reductase
VKORK	vitamin K 2,3-epoxide reductase complex
VT	venous thrombosis
VTE	venous thromboembolism
β_2_GPI	β_2_-glycopotein I

## References

[1] Liebson P.R. (2013). The Early History of Anticoagulants: 1915–1948. Hektoen Int. J. Med. Humanit..

[2] Milestones in Anticoagulant Drugs. https://www.hematology.org/about/history/50-years/milestones-anticoagulant-drugs.

[3] Fields W.S. (1991). The History of Leeching and Hirudin. Pathophysiol. Haemost. Thromb..

[4] Munshi Y., Ara I., Rafique H., Ahmad Z. (2008). Leeching in the History—A Review. Pak. J. Biol. Sci..

[5] Hsu E., Moosavi L. (2021). Biochemistry, Antithrombin III.

[6] Brouwers J.R.B.J., Roeters van Lennep J.E., Beinema M.J. (2019). Biosimilars of Low Molecular Weight Heparins: Relevant Background Information for Your Drug Formulary. Br. J. Clin. Pharmacol..

[7] Clivarin—Summary of the Product Characteristics. https://www.e-lactancia.org/media/papers/ReviparinClivarin-DS-SmPC2010.pdf.

[8] Imberti D., Marietta M., Polo Friz H., Cimminiello C. (2017). The Introduction of Biosimilars of Low Molecular Weight Heparins in Europe: A Critical Review and Reappraisal Endorsed by the Italian Society for Haemostasis and Thrombosis (SISET) and the Italian Society for Angiology and Vascular Medicine (SIAPAV). Thromb. J..

[9] Kataria B.C., Mehta D.S., Chhaiya S.B. (2013). Drug Lag for Cardiovascular Drug Approvals in India Compared with the US and EU Approvals. Indian Heart J..

[10] Troein P., Newton M., Scott K. The Impact of Biosimilar Competition in Europe. https://health.ec.europa.eu/system/files/2021-01/biosimilar_competition_en_0.pdf.

[11] Warkentin T.E., Greinacher A., Koster A. (2008). Bivalirudin. Thromb. Haemost..

[12] Enoxaparin—StatPearls. https://www.ncbi.nlm.nih.gov/books/NBK539865/.

[13] Fan P., Gao Y., Zheng M., Xu T., Schoenhagen P., Jin Z. (2018). Recent Progress and Market Analysis of Anticoagulant Drugs. J. Thorac. Dis..

[14] Abdulrehman J., Eikelboom J.W., Siegal D.M. (2019). Andexanet Alfa for Reversal of Factor Xa Inhibitors: A Critical Review of the Evidence. Future Cardiol..

[15] Burness C.B. (2015). Idarucizumab: First Global Approval. Drugs.

[16] Favaloro E.J., Pasalic L., Curnow J., Lippi G. (2017). Laboratory Monitoring or Measurement of Direct Oral Anticoagulants (DOACs): Advantages, Limitations and Future Challenges. Curr. Drug Metab..

[17] Petitou M., Casu B., Lindahl U. (2003). 1976–1983, a Critical Period in the History of Heparin: The Discovery of the Antithrombin Binding Site. Biochimie.

[18] Montinari M.R., Minelli S., De Caterina R. (2021). Eighty Years of Oral Anticoagulation: Learning from History. Vasc. Pharmacol..

[19] Bielecki S., Lee D., Hamad B. (2018). The Market for Oral Anticoagulants. Nat. Rev. Drug Discov..

[20] Onishi A., St Ange K., Dordick J.S., Linhardt R.J. (2016). Heparin and Anticoagulation. Front. Biosci. (Landmark Ed.).

[21] Global Anticoagulants Market (2021–2026) by Drug Class, Route of Administration, Geography and the Impact of COVID-19 with Ansoff Analysis. https://www.researchandmarkets.com/reports/5317270/global-anticoagulants-market-2021-2026-by-drug.

[22] Anticoagulant Reversal Drugs Market Size Report, 2020–2027. https://www.grandviewresearch.com/industry-analysis/anticoagulant-reversal-drugs-market.

[23] Cardenas J.C., Rein-Smith C.M., Church F.C. (2016). Overview of Blood Coagulation and the Pathophysiology of Blood Coagulation Disorders. Encyclopedia of Cell Biology.

[24] Versteeg H.H., Heemskerk J.W.M., Levi M., Reitsma P.H. (2013). New Fundamentals in Hemostasis. Physiol. Rev..

[25] Ashorobi D., Ameer M.A., Fernandez R. Thrombosis. StatPearls [Internet]. https://www.ncbi.nlm.nih.gov/books/NBK538430/.

[26] Christos S., Naples R. (2016). Anticoagulation Reversal and Treatment Strategies in Major Bleeding: Update 2016. West. J. Emerg. Med..

[27] Günther A., Ruppert C. (2006). Anticoagulants. Encyclopedia of Respiratory Medicine.

[28] Frederick R., Pochet L., Charlier C., Masereel B. (2012). Modulators of the Coagulation Cascade: Focus and Recent Advances in Inhibitors of Tissue Factor, Factor VIIa and Their Complex. Curr. Med. Chem..

[29] Becker R.C. (2021). Factor Xa Inhibitors: Critical Considerations for Clinical Development and Testing. J. Thromb. Thrombolysis.

[30] Munlemvo D.M. (2025). The Cell-Based Approach of Hemostasis, Coagulopathy, and the Implications for the Pediatric Cardiac Anesthesiologist. Front. Anesthesiol..

[31] Mackman N. (2012). New Insights into the Mechanisms of Venous Thrombosis. J. Clin. Investig..

[32] Neves A.R., Correia-Da-Silva M., Sousa E., Pinto M. (2016). Structure–Activity Relationship Studies for Multitarget Antithrombotic Drugs. Future Med. Chem..

[33] Patriota Y.B.G., Chaves L.L., Gocke E.H., Severino P., Soares M.F.R., Soares-Sobrinho J.L., Souto E.B., Taglietti M. (2021). Applied Nanotechnologies in Anticoagulant Therapy: From Anticoagulants to Coagulation Test Performance of Drug Delivery Systems. Appl. Nano.

[34] Walenga J.M. (2002). An Overview of the Direct Thrombin Inhibitor Argatroban. Pathophysiol. Haemost. Thromb..

[35] Acostamadiedo J.M., Lyer U.G., Owen J. (2005). Danaparoid Sodium. Expert Opin. Pharmacother..

[36] Wilde M.I., Markham A. (1997). Danaparoid. A Review of Its Pharmacology and Clinical Use in the Management of Heparin-Induced Thrombocytopenia. Drugs.

[37] Garcia D.A., Baglin T.P., Weitz J.I., Samama M.M. (2012). Parenteral Anticoagulants: Antithrombotic Therapy and Prevention of Thrombosis, 9th Ed: American College of Chest Physicians Evidence-Based Clinical Practice Guidelines. Chest.

[38] Gray E., Mulloy B., Barrowcliffe T.W. (2008). Heparin and Low-Molecular-Weight Heparin. Thromb. Haemost..

[39] Anticoagulation Products (LMWHs, Ultra LMWH, Direct Thrombin Inhibitors). https://hemonc.medicine.ufl.edu/files/2013/07/AnticoagRx.pdf.

[40] Thomas O., Lybeck E., Strandberg K., Tynngåd N., Schött U. (2015). Monitoring Low Molecular Weight Heparins at Therapeutic Levels: Dose-Responses of, and Correlations and Differences between APTT, Anti-Factor Xa and Thrombin Generation Assays. PLoS ONE.

[41] Smith S.A., Travers R.J., Morrissey J.H. (2015). How It All Starts: Initiation of the Clotting Cascade. Crit. Rev. Biochem. Mol. Biol..

[42] DeWald T.A., Washam J.B., Becker R.C. (2018). Anticoagulants: Pharmacokinetics, Mechanisms of Action, and Indications. Neurosurg. Clin. N. Am..

[43] HEPARIN Injectable|MSF Medical Guidelines. https://medicalguidelines.msf.org/en/viewport/EssDr/english/heparin-injectable-16682769.html.

[44] Heparin Infusion Guidelines|Anticoagulation Services. https://depts.washington.edu/anticoag/home/content/heparin-infusion-guidelines.

[45] Guidelines for Heparin Dosage—Manchester University NHS Foundation Trust. https://mft.nhs.uk/the-trust/other-departments/laboratory-medicine/haematology/reference-ranges/#heparin.

[46] Leentjens J., Peters M., Esselink A.C., Smulders Y., Kramers C. (2017). Initial Anticoagulation in Patients with Pulmonary Embolism: Thrombolysis, Unfractionated Heparin, LMWH, Fondaparinux, or DOACs?. Br. J. Clin. Pharmacol..

[47] Park J., Byun Y. (2015). Recent Advances in Anticoagulant Drug Delivery. Expert Opin. Drug Deliv..

[48] Fragmin—Summary of Product Characteristics. https://www.medicines.org.uk/emc/product/4241/smpc/print.

[49] Fraxiparine—Summary of Product Characteristics. https://www.anm.ro/_/_RCP/rcp_2518_10.05.10.pdf.

[50] CHMP Lovenox—Summary of Product Characteristics. https://www.ema.europa.eu/en/documents/referral/lovenox-article-30-referral-annex-iii_en.pdf.

[51] INNOHEP—Résumé Des Caractéristiques Du Produit. http://agence-prd.ansm.sante.fr/php/ecodex/rcp/R0287004.htm.

[52] Geerts W.H., Heit J.A., Clagett G.P., Pineo G.F., Colwell C.W., Anderson J., Wheeler H.B. (2001). Prevention of Venous Thromboembolism. Chest.

[53] (2004). DVT and Pulmonary Embolism: Part II. Treatment and Prevention. American Family Physician.

[54] CHMP Arixtra—Annex I Summary of Product Characteristics. https://www.ema.europa.eu/en/documents/product-information/arixtra-epar-product-information_en.pdf.

[55] Ufer M. (2005). Comparative Pharmacokinetics of Vitamin K Antagonists: Warfarin, Phenprocoumon and Acenocoumarol. Clin. Pharmacokinet..

[56] Haustein K.O. (1999). Pharmacokinetic and Pharmacodynamic Properties of Oral Anticoagulants, Especially Phenprocoumon. Semin. Thromb. Hemost..

[57] Zirlik A., Bode C. (2017). Vitamin K Antagonists: Relative Strengths and Weaknesses vs. Direct Oral Anticoagulants for Stroke Prevention in Patients with Atrial Fibrillation. J. Thromb. Thrombolysis.

[58] Saha N. (2018). Clinical Pharmacokinetics and Drug Interactions. Pharmaceutical Medicine and Translational Clinical Research.

[59] CHMP Xarelto—Summary of Product Characteristics. https://www.ema.europa.eu/en/documents/product-information/xarelto-epar-product-information_en.pdf.

[60] CHMP Eliquis—Summary of Product Characteristics. https://www.ema.europa.eu/en/documents/product-information/eliquis-epar-product-information_en.pdf.

[61] Zibor—Summary of Product Characteristics. https://assets.hpra.ie/products/Human/27479/Licence_PA25279-001-001_10012025162847.pdf.

[62] Walenga J.M., Lyman G.H. (2013). Evolution of Heparin Anticoagulants to Ultra-Low-Molecular-Weight Heparins: A Review of Pharmacologic and Clinical Differences and Applications in Patients with Cancer. Crit. Rev. Oncol. Hematol..

[63] Danaparoid Sodium 750 Anti-Xa Units/0.6 Ml, Solution for Injection—Summary of Product Characteristics (SmPC)–(Emc). https://www.medicines.org.uk/emc/product/13201/smpc#gref.

[64] Ibbotson T., Perry C.M. (2002). Danaparoid: A Review of Its Use in Thromboembolic and Coagulation Disorders. Drugs.

[65] CHMP Elmiron—Annex I Summary of Product Characteristics. https://www.ema.europa.eu/en/documents/product-information/elmiron-epar-product-information_en.pdf.

[66] FDA Argatroban—Highlights of Prescribing Information. https://www.accessdata.fda.gov/drugsatfda_docs/label/2021/212035s000lbl.pdf.

[67] FDA Iprivask—Highlights of Prescribing Information. https://www.accessdata.fda.gov/drugsatfda_docs/label/2014/021271s006lbl.pdf.

[68] CHMP Refludan—Annex I Summary of Product Characteristics. https://www.ema.europa.eu/en/documents/product-information/refludan-epar-product-information_en.pdf.

[69] CHMP Angiox—Annex I Summary of Product Characteristics. https://www.ema.europa.eu/en/documents/product-information/angiox-epar-product-information_en.pdf.

[70] Pradaxa—Summary of Product Characteristics I. https://www.ema.europa.eu/en/documents/product-information/pradaxa-epar-product-information_en.pdf.

[71] CHMP Roteas—Summary of Product Characteristics. https://www.ema.europa.eu/en/documents/product-information/roteas-epar-product-information_en.pdf.

[72] FDA Bevyxxa—Highlights of Prescribing Information. https://www.accessdata.fda.gov/drugsatfda_docs/label/2017/208383s000lbl.Pdf.

[73] Ageno W., Gallus A.S., Wittkowsky A., Crowther M., Hylek E.M., Palareti G. (2012). Oral Anticoagulant Therapy: Antithrombotic Therapy and Prevention of Thrombosis, 9th Ed: American College of Chest Physicians Evidence-Based Clinical Practice Guidelines. Chest.

[74] van Miert J.H.A., Veeger N.J.G.M., Ten Cate-Hoek A.J., Piersma-Wichers M., Meijer K. (2020). Effect of Switching from Acenocoumarol to Phenprocoumon on Time in Therapeutic Range and INR Variability: A Cohort Study. PLoS ONE.

[75] Kumar P., Kapoor P. (2019). Meenu Acenocoumarol’s Pharmacokinetic: Linear or Not?. EXCLI J..

[76] Ferri N., Colombo E., Tenconi M., Baldessin L., Corsini A. (2022). Drug-Drug Interactions of Direct Oral Anticoagulants (DOACs): From Pharmacological to Clinical Practice. Pharmaceutics.

[77] Alquwaizani M., Buckley L., Adams C., Fanikos J. (2013). Anticoagulants: A Review of the Pharmacology, Dosing, and Complications. Curr. Emerg. Hosp. Med. Rep..

[78] Torri G., Naggi A. (2016). Heparin Centenary—An Ever-Young Life-Saving Drug. Int. J. Cardiol..

[79] Santos G.R.C., Tovar A.M.F., Capillé N.V.M., Pereira M.S., Pomin V.H., Mourão P.A.S. (2014). Structural and Functional Analyses of Bovine and Porcine Intestinal Heparins Confirm They Are Different Drugs. Drug Discov. Today.

[80] Stratta P., Karvela E., Canavese C., Quaglia M., Lazzarich E., Fenoglio R., Pergolini P., Bellomo G., Cena T., Magnani C. (2009). Structure-Activity Relationships of Low Molecular Weight Heparins Expose to the Risk of Achieving Inappropriate Targets in Patients with Renal Failure. Curr. Med. Chem..

[81] Weitz J.I. (1997). Low-Molecular-Weight Heparins. N. Engl. J. Med..

[82] Paolucci F., Claviés M.C., Donat F., Necciari J. (2002). Fondaparinux Sodium Mechanism of Action: Identification of Specific Binding to Purified and Human Plasma-Derived Proteins. Clin. Pharmacokinet..

[83] Dawes J. (1993). Interactions of Heparins in the Vascular Environment. Pathophysiol. Haemost. Thromb..

[84] Cassinelli G., Naggi A. (2016). Old and New Applications of Non-Anticoagulant Heparin. Int. J. Cardiol..

[85] McRae H.L., Militello L., Refaai M.A. (2021). Updates in Anticoagulation Therapy Monitoring. Biomedicines.

[86] Dorgalaleh A., Beigi P., Pakjoo M., Eslami M., Kiyamehr P., Khaseb S., Seifpour S., Tabibian S., Naderi M., Dabbagh A. (2019). Anticoagulation Therapy in Iran. Ann. Blood.

[87] Li B., Zhao H., Yu M. (2021). Techniques for Detection of Clinical Used Heparins. Int. J. Anal. Chem..

[88] Linkins L.A., Dans A.L., Moores L.K., Bona R., Davidson B.L., Schulman S., Crowther M. (2012). Treatment and Prevention of Heparin-Induced Thrombocytopenia: Antithrombotic Therapy and Prevention of Thrombosis, 9th Ed: American College of Chest Physicians Evidence-Based Clinical Practice Guidelines. Chest.

[89] Galanti K., Di Marino M., Mansour D., Testa S., Rossi D., Scollo C., Magnano R., Pezzi L., D’Alleva A., Forlani D. (2024). Current Antithrombotic Treatments for Cardiovascular Diseases: A Comprehensive Review. Rev. Cardiovasc. Med..

[90] Sanchez-Holgado M., Sampedro M., Zozaya C., Permuy Romero C., Alvarez-Garcia P., La Banda-Montalvo L., Nieto C., Pellicer A. (2024). Bemiparin in Neonatal Thrombosis: Therapeutic Dosing and Safety. J. Perinatol..

[91] Del Bono R., Martini G., Volpi R. (2011). Update on Low Molecular Weight Heparins at the Beginning of Third Millennium. Focus on Reviparin. Eur. Rev. Med. Pharmacol. Sci..

[92] Piazza G., Goldhaber S.Z. (2006). Acute Pulmonary Embolism—Part II: Treatment and Prophylaxis. Circulation.

[93] Senturk A., Ucar E.Y., Berk S., Ozlu T., Altlnsoy B., Dabak G., Caklr E., Kadloglu E.E., Sen H.S., Ozsu S. (2015). Should Low-Molecular-Weight Heparin Be Preferred Over Unfractionated Heparin After Thrombolysis for Severity Pulmonary Embolism?. Clin. Appl. Thromb..

[94] Lazrak H.H., René É., Elftouh N., Leblanc M., Lafrance J.P. (2017). Safety of Low-Molecular-Weight Heparin Compared to Unfractionated Heparin in Hemodialysis: A Systematic Review and Meta-Analysis. BMC Nephrol..

[95] Wong S.S.M., Lau W.Y., Chan P.K., Wan C.K., Cheng Y.L. (2016). Low-Molecular Weight Heparin Infusion as Anticoagulation for Haemodialysis. Clin. Kidney J..

[96] Hao C., Sun M., Wang H., Zhang L., Wang W. (2019). Low Molecular Weight Heparins and Their Clinical Applications. Prog. Mol. Biol. Transl. Sci..

[97] UW Medicine—Low Molecular Weight Heparin Dosing Recommendations. http://depts.washington.edu/anticoag/home/sites/default/files/LMWH_Dosing_Recommendations_March_2013.pdf.

[98] Bertoli E.D., Barros M.L.S., Pasqualotto E., Lima P.L.G., Camerotte R., Nienkötter T.F., Souza M.E.C., Floriano I.T., Kelly F.A. (2025). Enoxaparin Versus Unfractionated Heparin in Acute Coronary Syndrome Without St-Segment Elevation: A Systematic Review and Meta-Analysis. Int. J. Cardiovasc. Sci..

[99] Hochart H., Vincent Jenkins P., Smith O.P., White B. (2006). Low-Molecular Weight and Unfractionated Heparins Induce a Downregulation of Inflammation: Decreased Levels of Proinflammatory Cytokines and Nuclear Factor-ΚB in LPS-Stimulated Human Monocytes. Br. J. Haematol..

[100] Mulloy B., Hogwood J., Gray E., Lever R., Page C.P. (2016). Pharmacology of Heparin and Related Drugs. Pharmacol. Rev..

[101] Li Z., Li Z. (2025). Development of the Anticoagulant Drug Fondaparinux Sodium. Medicinal Chemistry and Drug Development.

[102] Bates S.M., Weitz J.I. (2003). Emerging Anticoagulant Drugs. Arterioscler. Thromb. Vasc. Biol..

[103] Harenberg J. (2010). Idraparinux and Idrabiotaparinux. Expert Rev. Clin. Pharmacol..

[104] Harenberg J., Vukojevic Y., Mikus G., Joerg I., Weiss C. (2008). Long Elimination Half-Life of Idraparinux May Explain Major Bleeding and Recurrent Events of Patients from the van Gogh Trials. J. Thromb. Haemost..

[105] Song Y., Li X., Pavithra S., Li D. (2013). Idraparinux or Idrabiotaparinux for Long-Term Venous Thromboembolism Treatment: A Systematic Review and Meta-Analysis of Randomized Controlled Trials. PLoS ONE.

[106] Büller H.R. (2011). Efficacy and Safety of Once Weekly Subcutaneous Idrabiotaparinux in the Treatment of Patients with Symptomatic Deep Venous Thrombosis. J. Thromb. Haemost..

[107] Weitz J.I. (2003). Heparan Sulfate: Antithrombotic or Not?. J. Clin. Investig..

[108] Kim H.N., Whitelock J.M., Lord M.S. (2017). Structure-Activity Relationships of Bioengineered Heparin/Heparan Sulfates Produced in Different Bioreactors. Molecules.

[109] Kong Y., Chen H., Wang Y.Q., Meng L., Wei J.F. (2014). Direct Thrombin Inhibitors: Patents 2002–2012 (Review). Mol. Med. Rep..

[110] Fareed J., Jeske W.P. (2004). Small-Molecule Direct Antithrombins: Argatroban. Best Pract. Res. Clin. Haematol..

[111] Frame J.N., Rice L., Bartholomew J.R., Whelton A. (2010). Rationale and Design of the PREVENT-HIT Study: A Randomized, Open-Label Pilot Study to Compare Desirudin and Argatroban in Patients with Suspected Heparin-Induced Thrombocytopenia with or without Thrombosis. Clin. Ther..

[112] Greinacher A., Lubenow N., Eichler P. (2003). Anaphylactic and Anaphylactoid Reactions Associated with Lepirudin in Patients with Heparin-Induced Thrombocytopenia. Circulation.

[113] Bain J., Meyer A. (2015). Comparison of Bivalirudin to Lepirudin and Argatroban in Patients with Heparin-Induced Thrombocytopenia. Am. J. Health Syst. Pharm..

[114] Schulman S. (2014). Advantages and Limitations of the New Anticoagulants. J. Intern. Med..

[115] Ruff C.T., Giugliano R.P., Braunwald E., Hoffman E.B., Deenadayalu N., Ezekowitz M.D., Camm A.J., Weitz J.I., Lewis B.S., Parkhomenko A. (2014). Comparison of the Efficacy and Safety of New Oral Anticoagulants with Warfarin in Patients with Atrial Fibrillation: A Meta-Analysis of Randomised Trials. Lancet.

[116] Robertson L., Kesteven P., Mccaslin J.E. (2015). Oral Direct Thrombin Inhibitors or Oral Factor Xa Inhibitors for the Treatment of Pulmonary Embolism. Cochrane Database Syst. Rev..

[117] Piazza G., Bikdeli B., Pandey A.K., Krishnathasan D., Khairani C.D., Bejjani A., Morrison R.H., Hogan H., Rashedi S., Pfeferman M. (2025). Apixaban for Extended Treatment of Provoked Venous Thromboembolism. N. Engl. J. Med..

[118] Abdul-Rahman T., Roy P., Herrera-Calderón R.E., Mueller-Gomez J.L., Lisbona-Buzali M., Ulusan S., Awuah W.A., Kuchma N., Mehta N., Agrawal A. (2025). Rivaroxaban to Reduce the Risk of Major Cardiovascular Events in Patients with Chronic Coronary Artery Disease or Peripheral Artery Disease: A Narrative Review. Expert Opin. Drug Saf..

[119] Barr D., Epps Q.J. (2019). Direct Oral Anticoagulants: A Review of Common Medication Errors. J. Thromb. Thrombolysis.

[120] Reilly P.A., Lehr T., Haertter S., Connolly S.J., Yusuf S., Eikelboom J.W., Ezekowitz M.D., Nehmiz G., Wang S., Wallentin L. (2014). The Effect of Dabigatran Plasma Concentrations and Patient Characteristics on the Frequency of Ischemic Stroke and Major Bleeding in Atrial Fibrillation Patients: The RE-LY Trial (Randomized Evaluation of Long-Term Anticoagulation Therapy). J. Am. Coll. Cardiol..

[121] Brighton T.A. (2004). The Direct Thrombin Inhibitor Melagatran/Ximelagatran. Med. J. Aust..

[122] Granger C.B., Alexander J.H., McMurray J.J.V., Lopes R.D., Hylek E.M., Hanna M., Al-Khalidi H.R., Ansell J., Atar D., Avezum A. (2011). Apixaban versus Warfarin in Patients with Atrial Fibrillation. N. Engl. J. Med..

[123] Patel M.R., Mahaffey K.W., Garg J., Pan G., Singer D.E., Hacke W., Breithardt G., Halperin J.L., Hankey G.J., Piccini J.P. (2011). Rivaroxaban versus Warfarin in Nonvalvular Atrial Fibrillation. N. Engl. J. Med..

[124] Giugliano R.P., Ruff C.T., Braunwald E., Murphy S.A., Wiviott S.D., Halperin J.L., Waldo A.L., Ezekowitz M.D., Weitz J.I., Špinar J. (2013). Edoxaban versus Warfarin in Patients with Atrial Fibrillation. N. Engl. J. Med..

[125] Warfarin—StatPearls. https://www.ncbi.nlm.nih.gov/books/NBK470313/.

[126] Marietta M., Coluccio V., Boriani G., Luppi M. (2020). Effects of Anti-Vitamin k Oral Anticoagulants on Bone and Cardiovascular Health. Eur. J. Intern. Med..

[127] Maniscalco L., Varello K., Zoppi S., Abbamonte G., Ferrero M., Torres E., Ostorero F., Rossi F., Bozzetta E. (2021). Abnormal Prothrombin (Pivka-ii) Expression in Canine Tissues as an Indicator of Anticoagulant Poisoning. Animals.

[128] Pengo V., Denas G. (2018). Optimizing Quality Care for the Oral Vitamin K Antagonists (VKAs). Hematol. Am. Soc. Hematol. Educ. Program.

[129] Kahn S.R. (2016). The Post-Thrombotic Syndrome. Hematol. Am. Soc. Hematol. Educ. Program.

[130] Al-Samkari H., Connors J.M. (2018). Dual Anticoagulation with Fondaparinux and Dabigatran for Treatment of Cancer-Associated Hypercoagulability. Am. J. Hematol..

[131] Shimizu K., Sasaki T., Tomaru T., Noike H. (2016). Extensive Deep Vein Thrombosis Treatment Using Fondaparinux and Edoxaban: A Case Report. Thromb. J..

[132] Kruger P.C., Eikelboom J.W., Douketis J.D., Hankey G.J. (2019). Deep Vein Thrombosis: Update on Diagnosis and Management. Med. J. Aust..

[133] Carroll B.J., Piazza G., Goldhaber S.Z. (2019). Sulodexide in Venous Disease. J. Thromb. Haemost..

[134] Kakkos S.K., Gohel M., Baekgaard N., Bauersachs R., Bellmunt-Montoya S., Black S.A., Ten Cate-Hoek A.J., Elalamy I., Enzmann F.K., Geroulakos G. (2021). Clinical Practice Guideline Document Editor’s Choice-European Society for Vascular Surgery (ESVS) 2021 Clinical Practice Guidelines on the Management of Venous Thrombosis. Eur. J. Vasc. Endovasc. Surg..

[135] Haiey P.M. (2017). Overview of Venous Thromboembolism. Am. J. Manag. Care.

[136] Alkarithi G., Duval C., Shi Y., Macrae F.L., Ariëns R.A.S. (2021). Thrombus Structural Composition in Cardiovascular Disease. Arterioscler. Thromb. Vasc. Biol..

[137] Hepburn-Brown M., Darvall J., Hammerschlag G. (2019). Acute Pulmonary Embolism: A Concise Review of Diagnosis and Management. Intern. Med. J..

[138] Yoo H.H.B., Nunes-Nogueira V.S., Fortes Villas Boas P.J. (2020). Anticoagulant Treatment for Subsegmental Pulmonary Embolism. Cochrane Database Syst. Rev..

[139] Shrestha S., Coy S., Bekelis K. (2017). Oral Antiplatelet and Anticoagulant Agents in the Prevention and Management of Ischemic Stroke. Curr. Pharm. Des..

[140] Proietti M., Mairesse G.H., Goethals P., Scavee C., Vijgen J., Blankoff I., Vandekerckhove Y., Lip G.Y.H. (2017). Cerebrovascular Disease, Associated Risk Factors and Antithrombotic Therapy in a Population Screening Cohort: Insights from the Belgian Heart Rhythm Week Programme. Eur. J. Prev. Cardiol..

[141] Hart R.G., Pearce L.A., Aguilar M.I. (2007). Meta-Analysis: Antithrombotic Therapy to Prevent Stroke in Patients Who Have Nonvalvular Atrial Fibrillation. Ann. Intern. Med..

[142] Kapil N., Datta Y.H., Alakbarova N., Bershad E., Selim M., Liebeskind D.S., Bachour O., Rao G.H.R., Divani A.A. (2017). Antiplatelet and Anticoagulant Therapies for Prevention of Ischemic Stroke. Clin. Appl. Thromb..

[143] Murthy S.B., Gupta A., Merkler A.E., Navi B.B., Mandava P., Iadecola C., Sheth K.N., Hanley D.F., Ziai W.C., Kamel H. (2017). Restarting Anticoagulant Therapy After Intracranial Hemorrhage: A Systematic Review and Meta-Analysis. Stroke.

[144] Kang M., Alahmadi M., Sawh S., Kovacs M.J., Lazo-Langner A. (2015). Fondaparinux for the Treatment of Suspected Heparin-Induced Thrombocytopenia: A Propensity Score–Matched Study. Blood.

[145] Szyda Ł., Klosinska M.K., Poninska N.P., Mrozowska-Peruga E.M.P., Kasprzak J.D.K. (2021). Dabigatran as First-Line Treatment for Heparin-Induced Thrombocytopenia in the Intensive Cardiac Care Setting. Eur. Heart J..

[146] Carré J., Jourdi G., Gendron N., Helley D., Gaussem P., Darnige L. (2022). Recent Advances in Anticoagulant Treatment of Immune Thrombosis: A Focus on Direct Oral Anticoagulants in Heparin-Induced Thrombocytopenia and Anti-Phospholipid Syndrome. Int. J. Mol. Sci..

[147] He Y., He H., Liu D., Long Y., Su L., Cheng W. (2018). Fondaparinux in a Critically Ill Patient with Heparin-Induced Thrombocytopenia: A Case Report. Medicine.

[148] Linkins L.A., Hu G., Warkentin T.E. (2018). Systematic Review of Fondaparinux for Heparin-Induced Thrombocytopenia: When There Are No Randomized Controlled Trials. Res. Pract. Thromb. Haemost..

[149] Carré J., Guérineau H., Le Beller C., Mauge L., Huynh B., Nili R., Planquette B., Clauser S., Smadja D.M., Helley D. (2021). Direct Oral Anticoagulants as Successful Treatment of Heparin-Induced Thrombocytopenia: A Parisian Retrospective Case Series. Front. Med..

[150] Cohen H., Isenberg D.A. (2021). How I Treat Anticoagulant-Refractory Thrombotic Antiphospholipid Syndrome. Blood.

[151] Streiff M.B., Bockenstedt P.L., Cataland S.R., Chesney C., Eby C., Fanikos J., Fogarty P.F., Gao S., Garcia-Aguilar J., Goldhaber S.Z. (2011). Venous Thromboembolic Disease. J. Natl. Compr. Cancer Netw..

[152] Schrag D., Uno H., Rosovsky R., Rutherford C., Sanfilippo K., Villano J.L., Drescher M., Jayaram N., Holmes C., Feldman L. (2023). Direct Oral Anticoagulants vs Low-Molecular-Weight Heparin and Recurrent VTE in Patients with Cancer: A Randomized Clinical Trial. JAMA.

[153] Schlömmer C., Brandtner A., Bachler M. (2021). Antithrombin and Its Role in Host Defense and Inflammation. Int. J. Mol. Sci..

[154] Rochette L., Ghibu S. (2021). Mechanics Insights of Alpha-Lipoic Acid against Cardiovascular Diseases during COVID-19 Infection. Int. J. Mol. Sci..

[155] Thachil J., Tang N., Gando S., Falanga A., Cattaneo M., Levi M., Clark C., Iba T. (2022). ISTH Interim Guidance on Recognition and Management of Coagulopathy in COVID-19. J. Thromb. Haemost..

[156] Nab L., Visser C., van Bussel B.C.T., Beishuizen A., Bemelmans R.H.H., ten Cate H., Croles F.N., van Guldener C., de Jager C.P.C., Huisman M.V. (2025). Assessing Differential Application of Thromboprophylaxis Regimes Related to Risk of Pulmonary Embolism and Mortality in COVID-19 Patients through Instrumental Variable Analysis. Sci. Rep..

[157] Moores L.K., Tritschler T., Brosnahan S., Carrier M., Collen J.F., Doerschug K., Holley A.B., Jimenez D., Le Gal G., Rali P. (2020). Prevention, Diagnosis, and Treatment of VTE in Patients with Coronavirus Disease 2019: CHEST Guideline and Expert Panel Report. Chest.

[158] COVID-19: Hypercoagulability—UpToDate. https://www.uptodate.com/contents/covid-19-hypercoagulability?search=vtecovid&source=search_result&selectedTitle=1~150&usage_type=default&display_rank=1.

[159] Schutgens R.E. (2021). DOAC in COVID-19: Yes or No?. HemaSphere.

[160] Roguljić H., Arambašić J., Ninčević V., Kuna L., Šesto I., Tabll A., Smolić R., Včev A., Primorac D., Wu G.Y. (2022). The Role of Direct Oral Anticoagulants in the Era of COVID-19: Are Antiviral Therapy and Pharmacogenetics Limiting Factors?. Croat. Med. J..

[161] Spyropoulos A.C., Connors J.M., Douketis J.D., Goldin M., Hunt B.J., Kotila T.R., Lopes R.D., Schulman S. (2022). Good Practice Statements for Antithrombotic Therapy in the Management of COVID-19: Guidance from the SSC of the ISTH. J. Thromb. Haemost..

[162] Aursulesei V., Costache I.I. (2019). Anticoagulation in Chronic Kidney Disease: From Guidelines to Clinical Practice. Clin. Cardiol..

[163] Fanikos J., Burnett A.E., Mahan C.E., Dobesh P.P. (2017). Renal Function and Direct Oral Anticoagulant Treatment for Venous Thromboembolism. Am. J. Med..

[164] Su X., Yan B., Wang L., Lv J., Cheng H., Chen Y. (2021). Oral Anticoagulant Agents in Patients with Atrial Fibrillation and CKD: A Systematic Review and Pairwise Network Meta-Analysis. Am. J. Kidney Dis..

[165] Stanton B.E., Barasch N.S., Tellor K.B. (2017). Comparison of the Safety and Effectiveness of Apixaban versus Warfarin in Patients with Severe Renal Impairment. Pharmacotherapy.

[166] Leung K.C.W., MacRae J.M. (2019). Anticoagulation in CKD and ESRD. J. Nephrol..

[167] Al-Shaer M.H., Ibrahim T. (2015). Safety and Efficacy of Fondaparinux in Renal Impairment. J. Pharm. Technol..

[168] Lisman T., Kleiss S., Patel V.C., Fisher C., Adelmeijer J., Bos S., Singanayagam A., Stoy S.H., Shawcross D.L., Bernal W. (2018). In Vitro Efficacy of Pro- and Anticoagulant Strategies in Compensated and Acutely Ill Patients with Cirrhosis. Liver Int..

[169] Qamar A., Vaduganathan M., Greenberger N.J., Giugliano R.P. (2018). Oral Anticoagulation in Patients with Liver Disease. J. Am. Coll. Cardiol..

[170] Ogresta D., Mrzljak A., Berkovic M.C., Bilic-Curcic I., Stojsavljevic-Shapeski S., Virovic-Jukic L. (2022). Coagulation and Endothelial Dysfunction Associated with NAFLD: Current Status and Therapeutic Implications. J. Clin. Transl. Hepatol..

[171] O’Leary J.G., Greenberg C.S., Patton H.M., Caldwell S.H. (2019). AGA Clinical Practice Update: Coagulation in Cirrhosis. Gastroenterology.

[172] Shah S.J., Singer D.E., Fang M.C., Reynolds K., Go A.S., Eckman M.H. (2019). Net Clinical Benefit of Oral Anticoagulation among Older Adults with Atrial Fibrillation. Circ. Cardiovasc. Qual. Outcomes.

[173] Giustozzi M., Castellucci L.A., Barnes G.D. (2020). Management of Anticoagulant Treatment and Anticoagulation-Related Complications in Nonagenarians. Hamostaseologie.

[174] Chao T.F., Liu C.J., Lin Y.J., Chang S.L., Lo L.W., Hu Y.F., Tuan T.C., Liao J.N., Chung F.P., Chen T.J. (2018). Oral Anticoagulation in Very Elderly Patients with Atrial Fibrillation. Circulation.

[175] Rutherford O.C.W., Jonasson C., Ghanima W., Söderdahl F., Halvorsen S. (2022). Effectiveness and Safety of Oral Anticoagulants in Elderly Patients with Atrial Fibrillation. Heart.

[176] Toader D.M. (2019). Treating Atrial Fibrillation in Very Old Patients with New Oral Anticoagulation Drugs: Arguments pro and Contra. Eur. J. Cardiol. Pract..

[177] Yarrington C.D., Valente A.M., Economy K.E. (2015). Cardiovascular Management in Pregnancy. Circulation.

[178] Alshawabkeh L., Economy K.E., Valente A.M. (2016). Anticoagulation During Pregnancy Evolving Strategies with a Focus on Mechanical Valves. J. Am. Coll. Cardiol..

[179] Liu T., Zhang L., Joo D., Sun S.C. (2017). NF-ΚB Signaling in Inflammation. Signal Transduct. Target. Ther..

[180] Olczyk P., Mencner Ł., Komosinska-Vassev K. (2015). Diverse Roles of Heparan Sulfate and Heparin in Wound Repair. Biomed. Res. Int..

[181] Galvan L. (1996). Effects of Heparin on Wound Healing. J. Wound Ostomy Cont. Nurs..

[182] Vijayakumar C., Prabhu R., Senthil Velan M., Muthu Krishnan V., Kalaiarasi R., T S. (2018). Role of Heparin Irrigation in the Management of Superficial Burns with Special Reference to Pain Relief and Wound Healing: A Pilot Study. Cureus.

[183] Hsu F.M., Hu M.H., Jiang Y.S., Lin B.Y., Hu J.J., Jan J.S. (2020). Antibacterial Polypeptide/Heparin Composite Hydrogels Carrying Growth Factor for Wound Healing. Mater. Sci. Eng. C. Mater. Biol. Appl..

[184] Manzoor S., Khan F.A., Muhammad S., Qayyum R., Muhammad I., Nazir U., Bashir M.M. (2019). Comparative Study of Conventional and Topical Heparin Treatment in Second Degree Burn Patients for Burn Analgesia and Wound Healing. Burns.

[185] Andrgie A.T., Darge H.F., Mekonnen T.W., Birhan Y.S., Hanurry E.Y., Chou H.Y., Wang C.F., Tsai H.C., Yang J.M., Chang Y.H. (2020). Ibuprofen-Loaded Heparin Modified Thermosensitive Hydrogel for Inhibiting Excessive Inflammation and Promoting Wound Healing. Polymers.

[186] Peng J., Zhao H., Tu C., Xu Z., Ye L., Zhao L., Gu Z., Zhao D., Zhang J., Feng Z. (2020). In Situ Hydrogel Dressing Loaded with Heparin and Basic Fibroblast Growth Factor for Accelerating Wound Healing in Rat. Mater. Sci. Eng. C. Mater. Biol. Appl..

[187] Durmaz C.E., Ozkan A., Senel B., Uyar H.A. (2012). Comparison of Effects of Unfractionated Heparin and Low Molecular Weight Heparin on Skin Wound Healing of Rats. Acta Cir. Bras..

[188] Cifuentes A., Gómez-Gil V., Ortega M.A., Asúnsolo Á., Coca S., Román J.S., Álvarez-Mon M., Buján J., García-Honduvilla N. (2020). Chitosan Hydrogels Functionalized with Either Unfractionated Heparin or Bemiparin Improve Diabetic Wound Healing. Biomed. Pharmacother..

[189] Solak B. (2016). Low-Molecular-Weight Heparins as Immunomodulators in Dermatology Practice. Am. J. Ther..

[190] Çevirme D., Savluk Ö.F., Başaran E.K., Aksoy R., Elibol A., Baş T., Keser S., Adademir T., Yilmaz B. (2020). Effects of Anticoagulant Drugs on Wound Healing Process in a Rat Model: A Comparative Study. J. Wound Care.

[191] Kraft C.T., Bellile E., Baker S.R., Kim J.C., Moyer J.S. (2015). Anticoagulant Complications in Facial Plastic and Reconstructive Surgery. JAMA Facial Plast. Surg..

[192] Hippensteel J.A., LaRiviere W.B., Colbert J.F., Langouët-Astrié C.J., Schmidt E.P. (2020). Heparin as a Therapy for COVID-19: Current Evidence and Future Possibilities. Am. J. Physiol.—Lung Cell. Mol. Physiol..

[193] Chis A.A., Arseniu A.M., Morgovan C., Dobrea C.M., Frum A., Juncan A.M., Butuca A., Ghibu S., Gligor F.G., Rus L.L. (2022). Biopolymeric Prodrug Systems as Potential Antineoplastic Therapy. Pharmaceutics.

[194] Li Q., Gan L., Tao H., Wang Q., Ye L., Zhang A., Feng Z. (2016). The Synthesis and Application of Heparin-Based Smart Drug Carrier. Carbohydr. Polym..

[195] Andrgie A.T., Birhan Y.S., Mekonnen T.W., Hanurry E.Y., Darge H.F., Lee R.H., Chou H.Y., Tsai H.C. (2019). Redox-Responsive Heparin–Chlorambucil Conjugate Polymeric Prodrug for Improved Anti-Tumor Activity. Polymers.

[196] Tong N.A.N., Tran N.Q., Nguyen X.T.D.T., Du Cao V., Nguyen T.P., Nguyen C.K. (2018). Thermosensitive Heparin-Pluronic^®^ Copolymer as Effective Dual Anticancer Drugs Delivery System for Combination Cancer Therapy. Int. J. Nanotechnol..

[197] Li J., Pan H., Qiao S., Li Y., Wang J., Liu W., Pan W. (2019). The Utilization of Low Molecular Weight Heparin-Poloxamer Associated Laponite Nanoplatform for Safe and Efficient Tumor Therapy. Int. J. Biol. Macromol..

[198] Khan F., Tritschler T., Kimpton M., Wells P.S., Kearon C., Weitz J.I., Büller H.R., Raskob G.E., Ageno W., Couturaud F. (2021). Long-Term Risk for Major Bleeding During Extended Oral Anticoagulant Therapy for First Unprovoked Venous Thromboembolism: A Systematic Review and Meta-Analysis. Ann. Intern. Med..

[199] Burr N., Lummis K., Sood R., Kane J.S., Corp A., Subramanian V. (2017). Risk of Gastrointestinal Bleeding with Direct Oral Anticoagulants: A Systematic Review and Network Meta-Analysis. Lancet Gastroenterol. Hepatol..

[200] Xu W.-W., Hu S.-J., Wu T. (2017). Risk Analysis of New Oral Anticoagulants for Gastrointestinal Bleeding and Intracranial Hemorrhage in Atrial Fibrillation Patients: A Systematic Review and Network Meta-Analysis. J. Zhejiang Univ. Sci. B.

[201] Veltkamp R., Rizos T., Horstmann S. (2013). Intracerebral Bleeding in Patients on Antithrombotic Agents. Semin. Thromb. Hemost..

[202] Pallazola V.A., Kapoor R.K., Kapoor K., McEvoy J.W., Blumenthal R.S., Gluckman T.J. (2019). Anticoagulation Risk Assessment for Patients with Non-Valvular Atrial Fibrillation and Venous Thromboembolism: A Clinical Review. Vasc. Med..

[203] Parks A.L., Fang M.C. (2017). Scoring Systems for Estimating the Risk of Anticoagulant-Associated Bleeding. Semin. Thromb. Hemost..

[204] Wolfe Z., Khan S.U., Nasir F., Raghu Subramanian C., Lash B. (2018). A Systematic Review and Bayesian Network Meta-Analysis of Risk of Intracranial Hemorrhage with Direct Oral Anticoagulants. J. Thromb. Haemost..

[205] Wu T., Lv C., Wu L., Chen W., Lv M., Jiang S., Zhang J. (2022). Risk of Intracranial Hemorrhage with Direct Oral Anticoagulants: A Systematic Review and Meta-Analysis of Randomized Controlled Trials. J. Neurol..

[206] Crowther M., Cuker A. (2019). How Can We Reverse Bleeding in Patients on Direct Oral Anticoagulants?. Kardiol. Pol..

[207] Dhakal P., Rayamajhi S., Verma V., Gundabolu K., Bhatt V.R. (2017). Reversal of Anticoagulation and Management of Bleeding in Patients on Anticoagulants. Clin. Appl. Thromb. Hemost..

[208] Jevtic S.D., Morris A.M., Warkentin T.E., Pai M. (2021). Heparin-Induced Thrombocytopenia. Can. Med. Assoc. J..

[209] Ahmed I., Majeed A., Powell R. (2007). Heparin Induced Thrombocytopenia: Diagnosis and Management Update. Postgrad. Med. J..

[210] Bircher A.J., Harr T., Hohenstein L., Tsakiris D.A. (2006). Hypersensitivity Reactions to Anticoagulant Drugs: Diagnosis and Management Options. Allergy.

[211] Arepally G.M., Cines D.B. (2020). Pathogenesis of Heparin-Induced Thrombocytopenia. Transl. Res..

[212] Warkentin T.E. (2019). Laboratory Diagnosis of Heparin-Induced Thrombocytopenia. Int. J. Lab. Hematol..

[213] Nilius H., Kaufmann J., Cuker A., Nagler M. (2021). Comparative Effectiveness and Safety of Anticoagulants for the Treatment of Heparin-Induced Thrombocytopenia. Am. J. Hematol..

[214] Duewell B.E., Briski M.J., Feih J.T., Rinka J.R.G., Tawil J.N. (2021). Argatroban Versus Bivalirudin in the Treatment of Suspected or Confirmed Heparin-Induced Thrombocytopenia. J. Pharm. Pract..

[215] Warkentin T.E. (2019). High-Dose Intravenous Immunoglobulin for the Treatment and Prevention of Heparin-Induced Thrombocytopenia: A Review. Expert Rev. Hematol..

[216] Mohanty E., Nazir S., Sheppard J.A.I., Forman D.A., Warkentin T.E. (2019). High-Dose Intravenous Immunoglobulin to Treat Spontaneous Heparin-Induced Thrombocytopenia Syndrome. J. Thromb. Haemost..

[217] Dougherty J.A., Yarsley R.L. (2021). Intravenous Immune Globulin (IVIG) for Treatment of Autoimmune Heparin-Induced Thrombocytopenia: A Systematic Review. Ann. Pharmacother..

[218] Gonzalez-Delgado P., Fernandez J. (2016). Hypersensitivity Reactions to Heparins. Curr. Opin. Allergy Clin. Immunol..

[219] Hofmeier K.S., Bircher A.J. (2015). Hypersensitivity Reactions to Modern Antiplatelet and Anticoagulant Drugs. Allergo J. Int..

[220] Chan Y.C., Valenti D., Mansfield A.O., Stansby G. (2000). Warfarin Induced Skin Necrosis. Br. J. Surg..

[221] Nazarian R.M., Van Cott E.M., Zembowicz A., Duncan L.M. (2009). Warfarin-Induced Skin Necrosis. J. Am. Acad. Dermatol..

[222] Oz B.S., Asgun F., Oz K., Kuralay E., Tatar H. (2007). Warfarin-Induced Skin Necrosis after Open Heart Surgery Due to Protein S and C Deficiency. Heart Vessel..

[223] Chorzempa A., Tamis J., Simon C., Palazzo A., Leber R., Coven D., Hong M.K. (2013). Safety and Feasibility of Intra-Arterial Bivalirudin Bolus Administration during Primary Angioplasty. Coron. Artery Dis..

[224] Hasija S., Talwar S., Makhija N., Chauhan S., Malhotra P., Chowdhury U.K., Krishna N.S., Sharma G. (2018). Randomized Controlled Trial of Heparin Versus Bivalirudin Anticoagulation in Acyanotic Children Undergoing Open Heart Surgery. J. Cardiothorac. Vasc. Anesth..

[225] Carli G., Farsi A., Chiarini F., Lippolis D., Cortellini G. (2019). Hypersensitivity Reactions to Non-Vitamin K Oral Anticoagulants—A Review of Literature and Diagnostic Work-up Proposal. Eur. Ann. Allergy Clin. Immunol..

[226] DeBois W.J., Liu J., Elmer B., Ebrahimi H., Voevidko L., Lee L.Y., Krieger K.H., Isom W.W., Girardi L.N. (2006). Heparin Sensitivity Test for Patients Requiring Cardiopulmonary Bypass. J. Extracorpor. Technol..

[227] Lisman T., Thachil J. (2020). Differentiating Biochemical from Clinical Heparin Resistance in COVID-19. J. Thromb. Thrombolysis.

[228] Anderson J.A.M., Saenko E.L. (2002). Editorial I: Heparin Resistance. Br. J. Anaesth..

[229] Chen X., Liu Y., Furukawa N., Jin D.Y., Paul Savage G., Stafford D.W., Suhara Y., Williams C.M., Tie J.K. (2021). A Novel Vitamin K Derived Anticoagulant Tolerant to Genetic Variations of Vitamin K Epoxide Reductase. J. Thromb. Haemost..

[230] Oldenburg J., Müller C.R., Rost S., Watzka M., Bevans C.G. (2014). Comparative Genetics of Warfarin Resistance. Hamostaseologie.

[231] Cîmpan P.L., Chira R.I., Mocan M., Anton F.P., Farcaş A.D. (2019). Oral Anticoagulant Therapy-When Art Meets Science. J. Clin. Med..

[232] Orsi F.A., Annichino Bizzacchi J.M., De Paula E.V., Ozelo M.C., Langley M.R., Weck K.E. (2010). VKORC1 V66M Mutation in African Brazilian Patients Resistant to Oral Anticoagulant Therapy. Thromb. Res..

[233] Watzka M., Geisen C., Bevans C.G., Sittinger K., Spohn G., Rost S., Seifried E., Müller C.R., Oldenburg J. (2011). Thirteen Novel VKORC1 Mutations Associated with Oral Anticoagulant Resistance: Insights into Improved Patient Diagnosis and Treatment. J. Thromb. Haemost..

[234] Sobh M.M., Abdalbary M., Elnagar S., Nagy E., Elshabrawy N., Abdelsalam M., Asadipooya K., El-Husseini A. (2022). Secondary Osteoporosis and Metabolic Bone Diseases. J. Clin. Med..

[235] Márquez-Grant N., Baldini E., Jeynes V., Biehler-Gomez L., Aoukhiyad L., Passalacqua N.V., Giordano G., Di Candia D., Cattaneo C. (2022). How Do Drugs Affect the Skeleton? Implications for Forensic Anthropology. Biology.

[236] Signorelli S.S., Scuto S., Marino E., Giusti M., Xourafa A., Gaudio A. (2019). Anticoagulants and Osteoporosis. Int. J. Mol. Sci..

[237] Butler A.J., Eismont F.J. (2021). Effects of Anticoagulant Medication on Bone-Healing. JBJS Rev..

[238] Palmirotta R. (2022). Direct Oral Anticoagulants (DOAC): Are We Ready for a Pharmacogenetic Approach?. J. Pers. Med..

[239] Harder S., Thürmann P. (1996). Clinically Important Drug Interactions with Anticoagulants. An Update. Clin. Pharmacokinet..

[240] Korucu F.C., Senyigit E., Köstek O., Demircan N.C., Erdogan B., Uzunoglu S., Cicin I. (2018). A Retrospective Study on Potential Drug Interactions: A Single Center Experience. J. Oncol. Sci..

[241] Ollier C., Faaij R.A., Santoni A., Duvauchelle T., van Haard P.M.M., Schoemaker R.C., Cohen A.F., de Greef R., Burggraaf J. (2002). Absence of Interaction of Fondaparinux Sodium with Aspirin and Piroxicam in Healthy Male Volunteers. Clin. Pharmacokinet..

[242] Barbieri M.A., Cutroneo P.M., Baratelli C., Cicala G., Battaglia A., Santoro V., Andò G., Spina E. (2021). Adverse Drug Reactions with Oral Anticoagulants: Data from Sicilian Spontaneous Reporting System Database. J. Clin. Pharm. Ther..

[243] Mekaj Y.H., Mekaj A.Y., Duci S.B., Miftari E.I. (2015). New Oral Anticoagulants: Their Advantages and Disadvantages Compared with Vitamin K Antagonists in the Prevention and Treatment of Patients with Thromboembolic Events. Ther. Clin. Risk Manag..

[244] Nutescu E., Chuatrisorn I., Hellenbart E. (2011). Drug and Dietary Interactions of Warfarin and Novel Oral Anticoagulants: An Update. J. Thromb. Thrombolysis.

[245] Mannheimer B., Andersson M.L., Järnbert-pettersson H., Lindh J.D. (2016). The Effect of Carbamazepine on Warfarin Anticoagulation: A Register-Based Nationwide Cohort Study Involving the Swedish Population. J. Thromb. Haemost..

[246] Shrader S.P., Fermo J.D., Dzikowski A.L. (2004). Azithromycin and Warfarin Interaction. Pharmacotherapy.

[247] Wang M., Zeraatkar D., Obeda M., Lee M., Garcia C., Nguyen L., Agarwal A., Al-Shalabi F., Benipal H., Ahmad A. (2021). Drug-Drug Interactions with Warfarin: A Systematic Review and Meta-Analysis. Br. J. Clin. Pharmacol..

[248] Vazquez S.R. (2018). Drug-Drug Interactions in an Era of Multiple Anticoagulants: A Focus on Clinically Relevant Drug Interactions. Blood.

[249] Di Minno A., Frigerio B., Spadarella G., Ravani A., Sansaro D., Amato M., Kitzmiller J.P., Pepi M., Tremoli E., Baldassarre D. (2017). Old and New Oral Anticoagulants: Food, Herbal Medicines and Drug Interactions. Blood Rev..

[250] Matsumura Y., Yokota M., Yoshioka H., Shibata S., Ida S., Takiguchi Y. (1999). Acute Effects of Griseofulvin on the Pharmacokinetics and Pharmacodynamics of Warfarin in Rats. J. Int. Med. Res..

[251] Fahmi A.M., Abdelsamad O., Elewa H. (2016). Rifampin-Warfarin Interaction in a Mitral Valve Replacement Patient Receiving Rifampin for Infective Endocarditis: A Case Report. Springerplus.

[252] Ahmed S.N. (2008). Antiepileptic Drugs and Warfarin. Can. Med. Assoc. J..

[253] Holbrook A.M., Pereira J.A., Labiris R., McDonald H., Douketis J.D., Crowther M., Wells P.S. (2005). Systematic Overview of Warfarin and Its Drug and Food Interactions. Arch. Intern. Med..

[254] Moore N., Pollack C., Butkerait P. (2015). Adverse Drug Reactions and Drug– Drug Interactions with over-the-Counter NSAIDs. Ther. Clin. Risk Manag..

[255] Choi K.H., Kim A.J., Son I.J., Kim K.H., Kim K.B., Ahn H., Lee E.B. (2010). Risk Factors of Drug Interaction between Warfarin and Nonsteroidal Anti-Inflammatory Drugs in Practical Setting. J. Korean Med. Sci..

[256] Banfield C., O’Reilly R., Chan E., Rowland M. (1983). Phenylbutazone-Warfarin Interaction in Man: Further Stereochemical and Metabolic Considerations. Br. J. Clin. Pharmacol..

[257] Kaufman J.M., Fauver H.E. (1980). Potentiation of Warfarin by Trimethoprim-Sulfamethoxazole. Urology.

[258] Imai S., Kadomura S., Momo K., Kashiwagi H., Sato Y., Miyai T., Sugawara M., Takekuma Y. (2020). Comparison of Interactions between Warfarin and Cephalosporins with and without the N-Methyl-Thio-Tetrazole Side Chain. J. Infect. Chemother..

[259] Warfarin Drug Interactions—StatPearls. https://www.ncbi.nlm.nih.gov/books/NBK441964/.

[260] Lin J.H., Yamazaki M. (2003). Role of P-Glycoprotein in Pharmacokinetics: Clinical Implications. Clin. Pharmacokinet..

[261] Tan C.S.S., Lee S.W.H. (2021). Warfarin and Food, Herbal or Dietary Supplement Interactions: A Systematic Review. Br. J. Clin. Pharmacol..

[262] Yu C.-P., Yang M.-S., Hsu P.-W., Lin S.-P., Hou Y.-C. (2021). Bidirectional Influences of Cranberry on the Pharmacokinetics and Pharmacodynamics of Warfarin with Mechanism Elucidation. Nutrients.

[263] Heghes S.C., Vostinaru O., Mogosan C., Miere D., Iuga C.A., Filip L. (2022). Safety Profile of Nutraceuticals Rich in Coumarins: An Update. Front. Pharmacol..

[264] Cattanach A., Sibindi S. (2016). Warfarin, St John’s Wort and INR. Aust. Prescr..

[265] Niccolai C.S., Hicks R.W., Oertel L., Francis J.L. (2004). Unfractionated Heparin: Focus on a High-Alert Drug. Pharmacotherapy.

[266] Grissinger M.C., Hicks R.W., Keroack M.A., Marella W.M., Vaida A.J. (2010). Harmful Medication Errors Involving Unfractionated and Low-Molecular-Weight Heparin in Three Patient Safety Reporting Programs. Jt. Comm. J. Qual. Patient Saf..

[267] Vogel E.A., Billups S.J., Herner S.J., Delate T. (2016). Renal Drug Dosing: Effectiveness of Outpatient Pharmacist-Based vs. Prescriber-Based Clinical Decision Support Systems. Appl. Clin. Inform..

[268] Egger F., Targa F., Unterholzner I., Grant R.P., Herrmann M., Wiedermann C.J. (2016). Medication Error When Switching from Warfarin to Rivaroxaban Leading to Spontaneous Large Ecchymosis of the Abdominal and Chest Wall. Clin. Pract..

[269] Gschwind L., Rollason V., Lovis C., Boehlen F., Bonnabry P., Dayer P., Desmeules J.A. (2013). Identification and Weighting of the Most Critical “Real-Life” Drug-Drug Interactions with Acenocoumarol in a Tertiary Care Hospital. Eur. J. Clin. Pharmacol..

[270] Whitworth M.M., Haase K.K., Fike D.S., Bharadwaj R.M., Young R.B., MacLaughlin E.J. (2017). Utilization and Prescribing Patterns of Direct Oral Anticoagulants. Int. J. Gen. Med..

[271] Tran E., Duckett A., Fisher S., Bohm N. (2017). Appropriateness of Direct Oral Anticoagulant Dosing for Venous Thromboembolism Treatment. J. Thromb. Thrombolysis.

[272] Steinberg B.A., Shrader P., Thomas L., Ansell J., Fonarow G.C., Gersh B.J., Kowey P.R., Mahaffey K.W., Naccarelli G., Reiffel J. (2016). Off-Label Dosing of Non-Vitamin K Antagonist Oral Anticoagulants and Adverse Outcomes: The ORBIT-AF II Registry. J. Am. Coll. Cardiol..

[273] Davidson B.L., Verheijen S., Lensing A.W.A., Gebel M., Brighton T.A., Lyons R.M., Rehm J., Prins M.H. (2014). Bleeding Risk of Patients with Acute Venous Thromboembolism Taking Nonsteroidal Anti-Inflammatory Drugs or Aspirin. JAMA Intern. Med..

[274] Fitzgerald J.L., Howes L.G. (2016). Drug Interactions of Direct-Acting Oral Anticoagulants. Drug Saf..

[275] Piran S., Schulman S., Panju M., Pai M. (2018). Oral Anticoagulant Dosing, Administration, and Storage: A Cross-Sectional Survey of Canadian Health Care Providers. J. Thromb. Thrombolysis.

[276] Henriksen J.N., Nielsen L.P., Hellebek A., Poulsen B.K. (2017). Medication Errors Involving Anticoagulants: Data from the Danish Patient Safety Database. Pharmacol. Res. Perspect..

[277] Horlocker T.T., Vandermeuelen E., Kopp S.L., Gogarten W., Leffert L.R., Benzon H.T. (2018). Regional Anesthesia in the Patient Receiving Antithrombotic or Thrombolytic Therapy: American Society of Regional Anesthesia and Pain Medicine Evidence-Based Guidelines (Fourth Edition). Reg. Anesth. Pain Med..

[278] Alrowily A., Jalal Z., Abutaleb M.H., Osman N.A., Alammari M., Paudyal V. (2021). Medication Errors Associated with Direct-Acting Oral Anticoagulants: Analysis of Data from National Pharmacovigilance and Local Incidents Reporting Databases. J. Pharm. Policy Pract..

[279] Haque H., Alrowily A., Jalal Z., Tailor B., Efue V., Sarwar A., Paudyal V. (2021). Direct Oral Anticoagulant-Related Medication Incidents and Pharmacists’ Interventions in Hospital in-Patients: Evaluation Using Reason’s Accident Causation Theory. Int. J. Clin. Pharm..

[280] Cravo E.N., Hays H.L., Badeti J., Spiller H.A., Rine N.I., Zhu M., Smith G.A. (2025). Therapeutic Errors Associated with Antithrombotic Medications Reported to United States Poison Centers. Am. J. Hematol..

[281] Sugrue A., Sanborn D., Amin M., Farwati M., Sridhar H., Ahmed A., Mehta R., Siontis K.C., Mulpuru S.K., Deshmukh A.J. (2021). Inappropriate Dosing of Direct Oral Anticoagulants in Patients with Atrial Fibrillation. Am. J. Cardiol..

[282] Zhang Z.X., van de Garde E.M.W., Söhne M., Harmsze A.M., van den Broek M.P.H. (2020). Quality of Clinical Direct Oral Anticoagulant Prescribing and Identification of Risk Factors for Inappropriate Prescriptions. Br. J. Clin. Pharmacol..

[283] Morgovan C., Dobrea C.M., Chis A.A., Juncan A.M., Arseniu A.M., Rus L.L., Gligor F.G., Ardelean S.A., Stoicescu L., Ghibu S. (2023). A Descriptive Analysis of Direct Oral Anticoagulant Drugs Dosing Errors Based on Spontaneous Reports from the EudraVigilance Database. Pharmaceuticals.

[284] Bruneau A., Schwab C., Anfosso M., Fernandez C., Hindlet P. (2019). Burden of Inappropriate Prescription of Direct Oral Anticoagulants at Hospital Admission and Discharge in the Elderly: A Prospective Observational Multicenter Study. Drugs Aging.

[285] Ferrat E., Fabre J., Galletout P., Boutin E., Le Breton J., Renard V., Frappé P., Bastuji-Garin S. (2021). Inappropriate Direct Oral Anticoagulant Prescriptions in Patients with Non-Valvular Atrial Fibrillation: Cross-Sectional Analysis of the French CACAO Cohort Study in Primary Care. Br. J. Gen. Pract..

[286] Greenhalgh D.L., Thomas W.A. (1990). Blackout during Cardiopulmonary Bypass. Anaesthesia.

[287] Breniaux M., Charpiat B. (2022). Analyse Des Pratiques de Prescription de l’héparine Sodique Au Pousse-Seringue Électrique. Ann. Pharm. Françaises.

[288] Arimura J., Poole R.L., Jeng M., Rhine W., Sharek P. (2008). Neonatal Heparin Overdose—A Multidisciplinary Team Approach to Medication Error Prevention. J. Pediatr. Pharmacol. Ther..

[289] Levito M.N., Coons J.C., Verrico M.M., Szymkowiak A., Legler B., Dueweke E.J., Kane-Gill S.L. (2021). A Systemwide Approach for Navigating the Dilemma of Oral Factor Xa Inhibitor Interference with Unfractionated Heparin Anti-Factor Xa Concentrations. Ann. Pharmacother..

[290] Bosma B.E., Hunfeld N.G.M., Roobol-Meuwese E., Dijkstra T., Coenradie S.M., Blenke A., Bult W., Melief P.H.G.J., Dixhoorn M.P.V., van den Bemt P.M.L.A. (2021). Voluntarily Reported Prescribing, Monitoring and Medication Transfer Errors in Intensive Care Units in The Netherlands. Int. J. Clin. Pharm..

[291] Dreijer A.R., Diepstraten J., Bukkems V.E., Mol P.G.M., Leebeek F.W.G., Kruip M.J.H.A., Van Den Bemt P.M.L.A. (2019). Anticoagulant Medication Errors in Hospitals and Primary Care: A Cross-Sectional Study. Int. J. Qual. Health Care.

[292] Gabriel M.A.M., Movilla R.O., Labián C.M., Mateos M.F.S., Salmador R.S. (2018). Enoxaparin Overdose in a Newborn. Arch. Argent. Pediatr..

[293] D’Souza A., Wu P., Jung L., Nungaray K., Richman M. (2019). Human-Centered Design of a Low Molecular Weight Heparin Order Set to Reduce Medication Errors. J. Healthc. Qual..

[294] Cooper T., White C.L., Taber D., Uber W.E., Kokko H., Mazur J. (2012). Safety and Effectiveness Outcomes of an Inpatient Collaborative Drug Therapy Management Service for Direct Thrombin Inhibitors. Am. J. Health Syst. Pharm..

[295] Farnan J.M. (2017). Situational Awareness and Patient Safety. AORN J..

[296] Ghibu S., Juncan A.M., Rus L.L., Frum A., Dobrea C.M., Chiş A.A., Gligor F.G., Morgovan C. (2021). The Particularities of Pharmaceutical Care in Improving Public Health Service during the COVID-19 Pandemic. Int. J. Environ. Res. Public Health.

[297] de Lorenzo-Pinto A., Ortega-Navarro C., Ribed A., Giménez-Manzorro Á., Ibáñez-García S., de Miguel-Guijarro Á., Ginel-Feito M.D., Herranz A., Sanjurjo-Sáez M. (2019). Cancellations of Elective Surgical Procedures Due to Inadequate Management of Chronic Medications. J. Clin. Pharm. Ther..

[298] Moia M., Squizzato A. (2020). Correction to: Reversal Agents for Oral Anticoagulant-Associated Major or Life-Threatening Bleeding. Intern. Emerg. Med..

[299] Andexanet Alfa—StatPearls. https://www.ncbi.nlm.nih.gov/books/NBK519499/.

[300] Thomas S., Makris M. (2018). The Reversal of Anticoagulation in Clinical Practice. Clin. Med..

[301] Protamine—StatPearls. https://www.ncbi.nlm.nih.gov/books/NBK547753/.

[302] Warfarin Toxicity—StatPearls. https://www.ncbi.nlm.nih.gov/books/NBK431112/.

[303] Levy J.H., Ageno W., Chan N.C., Crowther M., Verhamme P., Weitz J.I. (2016). When and How to Use Antidotes for the Reversal of Direct Oral Anticoagulants: Guidance from the SSC of the ISTH. J. Thromb. Haemost..

[304] Glund S., Stangier J., Schmohl M., Gansser D., Norris S., Van Ryn J., Lang B., Ramael S., Moschetti V., Gruenenfelder F. (2015). Safety, Tolerability, and Efficacy of Idarucizumab for the Reversal of the Anticoagulant Effect of Dabigatran in Healthy Male Volunteers: A Randomised, Placebo-Controlled, Double-Blind Phase 1 Trial. Lancet.

[305] Pollack C.V., Reilly P.A., Eikelboom J., Glund S., Verhamme P., Bernstein R.A., Dubiel R., Weitz J.I. (2015). Idarucizumab for Dabigatran Reversal. N. Engl. J. Med..

[306] Jaspers T., Shudofsky K., Huisman M.V., Meijer K., Khorsand N. (2021). A Meta-Analysis of Andexanet Alfa and Prothrombin Complex Concentrate in the Treatment of Factor Xa Inhibitor-Related Major Bleeding. Res. Pract. Thromb. Haemost..

[307] Patanwala A.E., Acquisto N.M., Erstad B.L. (2011). Prothrombin Complex Concentrate for Critical Bleeding. Ann. Pharmacother..

[308] Sparrow R.L., Simpson R.J., Greening D.W. (2017). A Protocol for the Preparation of Cryoprecipitate and Cryo-Depleted Plasma for Proteomic Studies. Methods Mol. Biol..

[309] Nascimento B., Goodnough L.T., Levy J.H. (2014). Cryoprecipitate Therapy. Br. J. Anaesth..

[310] Garcia D.A., Crowther M.A. (2012). Reversal of Warfarin: Case-Based Practice Recommendations. Circulation.

[311] HaileSelassie H., Befkadu E., Tayler B. (2018). Tillage Management of Bleeding in Patients Treated with Direct Oral Anticoagulants. US Pharmacop..

[312] Schultz N.H., Tran H.T.T., Bjørnsen S., Henriksson C.E., Sandset P.M., Holme P.A. (2017). The Reversal Effect of Prothrombin Complex Concentrate (PCC), Activated PCC and Recombinant Activated Factor VII against Anticoagulation of Xa Inhibitor. Thromb. J..

[313] Jourdi G., Delrue M., Stepanian A., Valaize J., Foulon-Pinto G., Demagny J., Duchemin J., Nedelec-Gac F., Darnige L., Curis E. (2019). Potential Usefulness of Activated Charcoal (DOAC Remove^®^) for DRVVT Testing in Patients Receiving Direct Oral AntiCoagulants. Thromb. Res..

[314] Exner T., Ahuja M., Ellwood L. (2019). Effect of an Activated Charcoal Product (DOAC Stop^TM^) Intended for Extracting DOACs on Various Other APTT-Prolonging Anticoagulants. Clin. Chem. Lab. Med..

[315] Milling T.J., Kaatz S. (2016). Preclinical and Clinical Data for Factor Xa and “Universal” Reversal Agents. Am. J. Med..

[316] Minghetti P., Cilurzo F., Franzé S., Musazzi U.M., Itri M. (2013). Low Molecular Weight Heparins Copies: Are They Considered to Be Generics or Biosimilars?. Drug Discov. Today.

[317] Drouet L. (2012). Low Molecular Weight Heparin Biosimilars: How Much Similarity for How Much Clinical Benefit?. Target. Oncol..

[318] Gupta R., Ponnusamy M.P. (2018). Analysis of Sulfates on Low Molecular Weight Heparin Using Mass Spectrometry: Structural Characterization of Enoxaparin. Expert Rev. Proteom..

[319] Li A., Li M.K., Crowther M., Vazquez S.R. (2020). Drug-Drug Interactions with Direct Oral Anticoagulants Associated with Adverse Events in the Real World: A Systematic Review. Thromb. Res..

[320] Bisio A., Urso E., Guerrini M., de Wit P., Torri G., Naggi A. (2017). Structural Characterization of the Low-Molecular-Weight Heparin Dalteparin by Combining Different Analytical Strategies. Molecules.

[321] Martínez González J., Monreal M., Ayani Almagia I., Llaudó Garín J., Ochoa Díaz De Monasterioguren L., Gutierro Adúriz I. (2018). Bioequivalence of a Biosimilar Enoxaparin Sodium to Clexane ^®^ after Single 100 Mg Subcutaneous Dose: Results of a Randomized, Double-Blind, Crossover Study in Healthy Volunteers. Drug Des. Dev. Ther..

[322] Kobbi Z., Kraiem H., Benlasfar Z., Marouani A., Massoud T., Boubaker S., Bouhaouala-Zahar B., Fenina N. (2017). Comparative Subcutaneous Repeated Toxicity Study of Enoxaparin Products in Rats. Regul. Toxicol. Pharmacol..

[323] Ramacciotti E., Ferreira U., Costa A.J.V., Raymundo S.R.O., Correa J.A., Neto S.G., Osvaldt A.B., Agati L., Aguiar V.C.R., Davila R. (2018). Efficacy and Safety of a Biosimilar Versus Branded Enoxaparin in the Prevention of Venous Thromboembolism Following Major Abdominal Surgery: A Randomized, Prospective, Single-Blinded, Multicenter Clinical Trial. Clin. Appl. Thromb. Hemost..

[324] Qneibi D., Ramacciotti E., Macedo A.S., Caffaro R.A., Agati L.B., Siddiqui F., Kouta A., Hoppensteadt D., Fareed J., Carter C.A. (2020). Comparative Studies on the Anticoagulant Profile of Branded Enoxaparin and a New Biosimilar Version. Clin. Appl. Thromb. Hemost..

[325] Mourier P.A.J., Guichard O.Y., Herman F., Viskov C. (2014). Isolation of a Pure Octadecasaccharide with Antithrombin Activity from an Ultra-Low-Molecular-Weight Heparin. Anal. Biochem..

[326] Nandurkar H., Chong B., Salem H., Gallus A., Ferro V., Mckinnon R. (2014). Low-Molecular-Weight Heparin Biosimilars: Potential Implications for Clinical Practice. Australian Low-Molecular-Weight Heparin Biosimilar Working Group (ALBW). Intern. Med. J..

[327] Heparina Sodica—Rezumatul Caracteristicilor Produsului. https://www.anm.ro/_/_RCP/RCP_7534_08.04.15.pdf.

[328] Favaloro E.J., Lippi G., Koutts J. (2011). Laboratory Testing of Anticoagulants: The Present and the Future. Pathology.

[329] Brisset A.C., Ferrández A., Krause M., Rathbun S., Marlar R., Korte W. (2016). The PiCT^®^ Test Is a Reliable Alternative to the Activated Partial Thromboplastin Time in Unfractionated Heparin Therapy Management: Results from a Multicenter Study. J. Thromb. Haemost..

[330] Samama M.M., Guinet C. (2011). Laboratory Assessment of New Anticoagulants. Clin. Chem. Lab. Med..

[331] Staley E.M., Simmons S.C., Feldman A.Z., Williams L.A., Pham H.P. (2019). Monitoring Fondaparinux in the Setting of Antithrombin Deficiency. Lab. Med..

[332] Ariano R.E., Bhattacharya S.K., Moon M., Brownell L.G. (2000). Failure of Danaparoid Anticoagulation for Cardiopulmonary Bypass. J. Thorac. Cardiovasc. Surg..

[333] Gitlin S.D., Deeb G.M., Yann C., Schmaier A.H. (1998). Intraoperative Monitoring of Danaparoid Sodium Anticoagulation during Cardiovascular Operations. J. Vasc. Surg..

[334] Wanat M.A., Hart S.R., Putney D., Liebl M.G., Chandler W. (2013). Alternative Monitoring of Argatroban Using Plasma-Diluted Thrombin Time. Ann. Pharmacother..

[335] Beiderlinden M., Werner P., Bahlmann A., Kemper J., Brezina T., Schäfer M., Görlinger K., Seidel H., Kienbaum P., Treschan T.A. (2018). Monitoring of Argatroban and Lepirudin Anticoagulation in Critically Ill Patients by Conventional Laboratory Parameters and Rotational Thromboelastometry—A Prospectively Controlled Randomized Double-Blind Clinical Trial. BMC Anesthesiol..

[336] Van Cott E.M., Roberts A.J., Dager W.E. (2017). Laboratory Monitoring of Parenteral Direct Thrombin Inhibitors. Semin. Thromb. Hemost..

[337] Zhang L., Long Y., Xiao H., Yang J., Toulon P., Zhang Z. (2018). Use of D-Dimer in Oral Anticoagulation Therapy. Int. J. Lab. Hematol..

[338] Dias J.D., Norem K., Doorneweerd D.D., Thurer R.L., Popovsky M.A., Omert L.A. (2015). Use of Thromboelastography (TEG) for Detection of New Oral Anticoagulants. Arch. Pathol. Lab. Med..

[339] Siddiqui F., Hoppensteadt D., Jeske W., Iqbal O., Tafur A., Fareed J. (2019). Factor Xa Inhibitory Profile of Apixaban, Betrixaban, Edoxaban, and Rivaroxaban Does Not Fully Reflect Their Biologic Spectrum. Clin. Appl. Thromb..

[340] Letertre L.R., Gudmundsdottir B.R., Francis C.W., Gosselin R.C., Skeppholm M., Malmstrom R.E., Moll S., Hawes E., Francart S., Onundarson P.T. (2016). A Single Test to Assay Warfarin, Dabigatran, Rivaroxaban, Apixaban, Unfractionated Heparin, and Enoxaparin in Plasma. J. Thromb. Haemost..

[341] Flanders M.M., Crist R., Rodgers G.M. (2003). Comparison of Five Thrombin Time Reagents. Clin. Chem..

[342] Samuelson B.T., Cuker A., Siegal D.M., Crowther M., Garcia D.A. (2017). Laboratory Assessment of the Anticoagulant Activity of Direct Oral Anticoagulants: A Systematic Review. Chest.

[343] Božič-Mijovski M., Malmströ R.E., Malovrh P., Antovic J.P., Vene N., Šinigoj P., Mavri A. (2016). Diluted Thrombin Time Reliably Measures Low to Intermediate Plasma Dabigatran Concentrations. Ann. Clin. Biochem..

[344] Kluft C., Meijer P., Kret R., Burggraaf J. (2013). Preincubation in the Prothrombinase-Induced Clotting Time Test (PiCT) Is Necessary for in Vitro Evaluation of Fondaparinux and to Be Avoided for the Reversible, Direct Factor Xa Inhibitor, Rivaroxaban. Int. J. Lab. Hematol..

[345] Hoppensteadt D., Walenga J.M., Cunanan J., Iqbal O., Fareed J. (2013). Prothrombinase Induced Clotting Time (PICT) and Commercially Available Diluted Russell’s Viper Venom Times For The Monitoring Of New Oral Anticoagulants. Blood.

[346] Pengo V., Bison E., Banzato A., Zoppellaro G., Jose S.P., Denas G. (2017). Lupus Anticoagulant Testing: Diluted Russell Viper Venom Time (DRVVT). Methods Mol. Biol..

[347] Newall F. (2013). Anti-Factor Xa (Anti-Xa) Assay. Methods Mol. Biol..

[348] Campbell P.J., Tirvengadum M.A., Pickering W., Cohen H., Ryan K.E. (1999). HEPTEST: A Suitable Method for Monitoring Heparin during Pregnancy. Clin. Lab. Haematol..

[349] Horton S., Augustin S. (2013). Activated Clotting Time (ACT). Methods Mol. Biol..

[350] Nowak G. (2003). The Ecarin Clotting Time, a Universal Method to Quantify Direct Thrombin Inhibitors. Pathophysiol. Haemost. Thromb..

[351] Vig S., Chitolie A., Bevan D., Halliday A., Dormandy J.A. (2022). Thromboelastography. Hematology.

[352] Kearon C., Akl E.A., Ornelas J., Blaivas A., Jimenez D., Bounameaux H., Huisman M., King C.S., Morris T.A., Sood N. (2016). Antithrombotic Therapy for VTE Disease: CHEST Guideline and Expert Panel Report. Chest.

[353] Legnani C., Martinelli I., Palareti G., Ciavarella A., Poli D., Ageno W., Testa S., Mastroiacovo D., Ciammaichella M., Bucherini E. (2019). D-Dimer Levels during and after Anticoagulation Withdrawal in Patients with Venous Thromboembolism Treated with Non-Vitamin K Anticoagulants. PLoS ONE.

[354] Babin J.L., Traylor K.L., Witt D.M. (2017). Laboratory Monitoring of Low-Molecular-Weight Heparin and Fondaparinux. Semin. Thromb. Hemost..

[355] Nguyen T.D., Nguyen T.N., Nguyen T.T.T., Ivanov I.A., Nguyen K.C., Tran Q.N., Hoang A.N., Utkin Y.N. (2019). Nanoencapsulation Enhances Anticoagulant Activity of Adenosine and Dipeptide IleTrp. Nanomaterials.

[356] Kar S., Sanderson H., Roy K., Benfenati E., Leszczynski J. (2022). Green Chemistry in the Synthesis of Pharmaceuticals. Chem. Rev..

[357] Dakshayani S.S., Marulasiddeshwara M.B., Sharath Kumar S.K., Ramesh G., Raghavendra Kumar P., Devaraja S., Hosamani R. (2019). Antimicrobial, Anticoagulant and Antiplatelet Activities of Green Synthesized Silver Nanoparticles Using Selaginella (Sanjeevini) Plant Extract. Int. J. Biol. Macromol..

[358] Lateef A., Folarin B.I., Oladejo S.M., Akinola P.O., Beukes L.S., Gueguim-Kana E.B. (2018). Characterization, Antimicrobial, Antioxidant, and Anticoagulant Activities of Silver Nanoparticles Synthesized from Petiveria Alliacea L. Leaf Extract. Prep. Biochem. Biotechnol..

[359] Kim H.S., Jun S.H., Koo Y.K., Cho S., Park Y. (2013). Green Synthesis and Nanotopography of Heparin-Reduced Gold Nanoparticles with Enhanced Anticoagulant Activity. J. Nanosci. Nanotechnol..

[360] Fredenburgh J.C., Weitz J.I. (2021). Factor XI as a Target for New Anticoagulants. Hamostaseologie.

[361] Hsu C., Hutt E., Bloomfield D.M., Gailani D., Weitz J.I. (2021). Factor XI Inhibition to Uncouple Thrombosis From Hemostasis: JACC Review Topic of the Week. J. Am. Coll. Cardiol..

[362] Mackman N., Bergmeier W., Stouffer G.A., Weitz J.I. (2020). Therapeutic Strategies for Thrombosis: New Targets and Approaches. Nat. Rev. Drug Discov..

[363] Liu T., Hashizume K., Krieg E., Chen H., Mukaida Y., Thelen K., Friedrichs F., Willmann S., Schwers S., Solms A. (2024). Pharmacokinetics, Pharmacodynamics, and Safety of Fesomersen, a Novel Antisense Inhibitor of Factor XI, in Healthy Chinese, Japanese, and Caucasian Volunteers. Clin. Transl. Sci..

[364] Verhamme P., Yi B.A., Segers A., Salter J., Bloomfield D., Büller H.R., Raskob G.E., Weitz J.I. (2021). Abelacimab for Prevention of Venous Thromboembolism. N. Engl. J. Med..

[365] Yi B.A., Freedholm D., Widener N., Wang X., Simard E., Cullen C., Al-Saady N.M., Lepor N.E., Coulter S., Lovern M. (2022). Pharmacokinetics and Pharmacodynamics of Abelacimab (MAA868), a Novel Dual Inhibitor of Factor XI and Factor XIa. J. Thromb. Haemost..

[366] Ruff C.T., Patel S.M., Giugliano R.P., Morrow D.A., Hug B., Kuder J.F., Goodrich E.L., Chen S.-A., Goodman S.G., Joung B. (2025). Abelacimab versus Rivaroxaban in Patients with Atrial Fibrillation. N. Engl. J. Med..

[367] Ieko M., Ohmura K., Naito S., Yoshida M., Kumano O. (2025). Development of New Anticoagulants Targeting Coagulation Factor XI and Prospects for Clinical Use. J. Cardiol..

[368] Nowotny B., Thomas D., Schwers S., Wiegmann S., Prange W., Yassen A., Boxnick S. (2022). First Randomized Evaluation of Safety, Pharmacodynamics, and Pharmacokinetics of BAY 1831865, an Antibody Targeting Coagulation Factor XI and Factor XIa, in Healthy Men. J. Thromb. Haemost..

[369] Weitz J.I., Bauersachs R., Becker B., Berkowitz S.D., Freitas M.C.S., Lassen M.R., Metzig C., Raskob G.E. (2020). Effect of Osocimab in Preventing Venous Thromboembolism Among Patients Undergoing Knee Arthroplasty: The FOXTROT Randomized Clinical Trial. JAMA.

[370] Thomas D., Thelen K., Kraff S., Schwers S., Schiffer S., Unger S., Yassen A., Boxnick S. (2019). BAY 1213790, a Fully Human IgG1 Antibody Targeting Coagulation Factor XIa: First Evaluation of Safety, Pharmacodynamics, and Pharmacokinetics. Res. Pract. Thromb. Haemost..

[371] Lorentz C.U., Verbout N.G., Wallisch M., Hagen M.W., Shatzel J.J., Olson S.R., Puy C., Hinds M.T., McCarty O.J.T., Gailani D. (2019). Contact Activation Inhibitor and Factor XI Antibody, AB023, Produces Safe, Dose-Dependent Anticoagulation in a Phase 1 First-In-Human Trial. Arterioscler. Thromb. Vasc. Biol..

[372] Kubitza D., Heckmann M., Distler J., Koechel A., Schwers S., Kanefendt F. (2022). Pharmacokinetics, Pharmacodynamics and Safety of BAY 2433334, a Novel Activated Factor XI Inhibitor, in Healthy Volunteers: A Randomized Phase 1 Multiple-Dose Study. Br. J. Clin. Pharmacol..

[373] Thomas D., Kanefendt F., Schwers S., Unger S., Yassen A., Boxnick S. (2021). First Evaluation of the Safety, Pharmacokinetics, and Pharmacodynamics of BAY 2433334, a Small Molecule Targeting Coagulation Factor XIa. J. Thromb. Haemost..

[374] Beale D., Dennison J., Boyce M., Mazzo F., Honda N., Smith P., Bruce M. (2021). ONO-7684 a Novel Oral FXIa Inhibitor: Safety, Tolerability, Pharmacokinetics and Pharmacodynamics in a First-in-Human Study. Br. J. Clin. Pharmacol..

[375] Chen R., Guan X., Hu P., Dong Y., Zhu Y., Zhang T., Zou J., Zhang S. (2022). First-In-Human Study to Assess the Safety, Pharmacokinetics, and Pharmacodynamics of SHR2285, a Small-Molecule Factor XIa Inhibitor in Healthy Subjects. Front. Pharmacol..

[376] Dilger A.K., Pabbisetty K.B., Corte J.R., De Lucca I., Fang T., Yang W., Pinto D.J.P., Wang Y., Zhu Y., Mathur A. (2022). Discovery of Milvexian, a High-Affinity, Orally Bioavailable Inhibitor of Factor XIa in Clinical Studies for Antithrombotic Therapy. J. Med. Chem..

[377] Campello E., Simioni P., Prandoni P., Ferri N. (2022). Clinical Pharmacology of Factor XI Inhibitors: New Therapeutic Approaches for Prevention of Venous and Arterial Thrombotic Disorders. J. Clin. Med..

[378] Ni S., Zhuo Z., Pan Y., Yu Y., Li F., Liu J., Wang L., Wu X., Li D., Wan Y. (2021). Recent Progress in Aptamer Discoveries and Modifications for Therapeutic Applications. ACS Appl. Mater. Interfaces.

[379] Chen K., Zhou J., Shao Z., Liu J., Song J., Wang R., Li J., Tan W. (2020). Aptamers as Versatile Molecular Tools for Antibody Production Monitoring and Quality Control. J. Am. Chem. Soc..

[380] Lee J.O., So H.M., Jeon E.K., Chang H., Won K., Kim Y.H. (2008). Aptamers as Molecular Recognition Elements for Electrical Nanobiosensors. Anal. Bioanal. Chem..

[381] Wakui K., Yoshitomi T., Yamaguchi A., Tsuchida M., Saito S., Shibukawa M., Furusho H., Yoshimoto K. (2019). Rapidly Neutralizable and Highly Anticoagulant Thrombin-Binding DNA Aptamer Discovered by MACE SELEX. Mol. Ther. Nucleic Acids.

[382] Troisi R., Napolitano V., Spiridonova V., Krauss I.R., Sica F. (2018). Several Structural Motifs Cooperate in Determining the Highly Effective Anti-Thrombin Activity of NU172 Aptamer. Nucleic Acids Res..

[383] De Fenza M., Eremeeva E., Troisi R., Yang H., Esposito A., Sica F., Herdewijn P., D’Alonzo D., Guaragna A. (2020). Structure–Activity Relationship Study of a Potent α-Thrombin Binding Aptamer Incorporating Hexitol Nucleotides. Chem. Eur. J..

[384] Cassinelli G., Torri G., Naggi A. (2020). Non-Anticoagulant Heparins as Heparanase Inhibitors. Adv. Exp. Med. Biol..

[385] Roy S., Lai H., Zouaoui R., Duffner J., Zhou H., Jayaraman L.P., Zhao G., Ganguly T., Kishimoto T.K., Venkataraman G. (2011). Bioactivity Screening of Partially Desulfated Low-Molecular-Weight Heparins: A Structure/Activity Relationship Study. Glycobiology.

[386] Pan Q., Zhang C., Wu X., Chen Y. (2020). Identification of a Heparosan Heptasaccharide as an Effective Anti-Inflammatory Agent by Partial Desulfation of Low Molecular Weight Heparin. Carbohydr. Polym..

